# Supplementary material for: Development of selection strategies for genetic improvement in production traits of Mecheri sheep based on a Bayesian multi trait evaluation
Source: PLoS One. 2023 Dec 14;18(12):e0289460. doi: 10.1371/journal.pone.0289460 (PMC10721189; doi:10.1371/journal.pone.0289460)
Supplement: S1 File — (PDF) [file pone.0289460.s001.pdf]

Lamb	Dam	Sire	BW	WW	SMW	NMW	YW
58	5	41	2.5	10.6	11.3	14.7	19
59	40	41	2.6	8.4	11.1	14.3	19
60	16	41	2.7	8.6	11.8	15.4	19
61	1	41	2.7	11.7	15.4	15.8	21
62	12	41	2.2	10.7	12.9	15.9	19
98	10	41	2.8	13.3	14	18.9	23
99	20	41	2.8	10.9	13.3	18.1	21
100	35	41	2.5	7.5	10.3	12	14
101	18	41	2.1	7.5	9.3	12.2	15
106	78	41	2.2	7.3	9.5		
107	8	41	2.7	8.4	11		
108	37	42	2.4	7.2	8.9		
109	82	42	2.2	9.7	13		
110	32	41	2.6	10.7	12.2	12.8	18
111	27	41	2.5	11.2	12.5	14.8	20
112	85	42	2.4	9.8	10.4	16.5	18
113	14	41	2.5	10.5	13.4	19.8	22
114	13	41	2.7	12.9	14	21	26
115	75	42	2.5	11.2	11.6	13.3	18
116	24	41	2.9	12.9	14.2	18.8	20
117	74	41	2	7.1			
118	36	41	2.8	9.3			
119	20	42	2.3	11.2	12.2	16.2	19
120	97	41	2.7	11.1	12.7	17	16
121	89	41	2.4	9.5	10.8	15.2	17
122	87	42	2.7	10.6	13.2	18	17
123	64	41	2	11.1	13.8	16.4	16
124	67	41	2.1	8.8	12.1	17	16
125	93	41	2.7	9.7	13.1	19.2	
126	66	41	2.2	5			
127	4	42	2.4	9.6	11.1	15.2	15
128	71	42	2.4				
129	1	41	2.8	10.8	11.2	15.4	18
130	88	42	2.4	7.8	10.6	14.7	14
131	91	41	2.8	9.3	10	16.2	21
132	69	42	2.8	10	11	15.3	18
133	95	41	2	7.8	8.6	14.8	17
134	18	42	2.6				
135	96	42	2.2	9.1	11.2	11.6	
136	2	42	2.5	11.4	13.7	14.5	18
137	83	42	2.2	10.7	11.8	12.7	
138	80	41	2	11	13.9		
139	26	41	2.6	13.6	16.8	16.8	20
140	34	41	2.9				
141	21	42	2.6	10.8	16.5		
142	15	41	2.3	8.9	11.2	11	
144	9	41	2.6	12.5	14.8	15.3	
143	33	41	2.3	9.3	12	12.2	
145	72	42	2.1	9.3	12	12.5	

146	30	42	2.4	10.7	12		
148	79	42	1.8	9.1	11.3	12.1	
147	92	42	2.3	10.5	11.2	11.2	
149	70	42	2.1	7.8	9.5		
150	7	42	2	11.7	14.5		
151	5	41	2.8	12.2	15.8	15.9	16
152	63	41	1.8	9.8	12		
153	35	42	2.2	10.8	12		
156	3	41	2.9	10	12.5	12	
154	23	41	2.5	12.5	13.8	13.5	16
155	28	41	2.8	8.5	10.8		
157	90	41	2.6	11.3	13.4	12.5	
158	22	42	2.8	12.7	14.4	14.4	15
159	68	41	2.4	11.2	13	12.2	
160	6	42	1.9	8.7	11.2	12	
161	65	41	2.4	12.4	12.9		
162	12	41	2.9	12.1	17.3	21.2	24
163	39	42	2.8	11.1	14		
164	16	42	2.6				
165	86	42	2.4				
166	84	42	1.7	9.7	10.8		
167	81	42	2	6.1			
168	29	41	3	11.1			
169	95	42	2.5	8.4			
170	8	41	2.6	10.2	11.9	14.1	
171	24	42	2.9	7.9			
172	37	41	2.9	7.5			
174	71	41	2	6.7			
173	75	41	2.4	8.8	9.7		
175	4	41	2.4	6.9			
176	27	41	2.3	8.1			
177	14	41	2.4	8			
178	19	41	2.8	7.6			
179	94	41	2.7	9.1	11.8		
181	18	41	2.6	10.5	14	14.2	
182	20	41	2.7	5.8			
184	97	42	2.7	8.3			
185	31	42	2.2	8.4	10.5		
186	78	41	2.5				
187	64	42	2.4				
188	76	41	1.3				
227	6	41	2.5	5.6			
228	55	42	2	5.8			
229	34	42	2.7	6.6			
233	17	41	2.5				
230	70	41	1.9				
231	92	41	2.2	10.1			
232	96	41	2.2	6.4			
234	80	42	2.6	10.5			
235	60	47	2.1	7.1	8.4		

236	83	41	2.3				
237	33	42	2.7	8.6			
240	81	41	2.4	9.9	11		
239	86	41	2.5	7.1			
241	3	42	2.5	9.2			
242	15	42	2.6	8.2	8.8		
243	72	41	2.6	9.2			
244	23	42	2.4	5.9			
245	28	42	2.8	8.8			
246	26	42	2.7	9.2			
247	48	41	2.3				
248	68	42	2.7	5.8			
249	52	42	2	5.3			
250	49	41	1.5				
252	7	41	2.4				
253	38	42	2.4				
254	89	42	1.7	6.2			
255	79	41	1.9	5.4			
256	30	45	3	7.9			
257	12	47	2.3	8.5			
258	22	45	2.3				
259	90	42	2.6	6.6			
260	13	45	2.6				
262	44	41	2.5				
261	69	41	2.1	5.6			
263	85	41	2.8	5.1			
264	5	42	2.6				
265	9	47	2.4	6			
266	21	41	1.9				
268	66	41	2.7				
267	93	41	2.5	5.8			
272	73	42	2.1	13.2	17	19	20
274	24	41	2.6	10.8	19.1	22.4	23
275	78	45	3	12.6	17.2	19.5	25
277	34	41	3	13.5	15.5	16.6	22
276	38	41	2.5	9.8	13.5	14.2	19
278	55	41	3	12.5	16.2	17.5	25
279	75	41	3	10.2	13.2	13.8	20
280	51	41	3	12.8	14.9	17.6	20
281	52	41	3	12	15.4	15.2	20
282	83	45	3	12	13.8	14.3	20
284	43	47	2.5	10.5	13.5	14.3	19
283	60	47	3	10.5	14.6	14.6	20
285	37	42	3.2	12.5	15.7	15.8	22
287	32	45	2.9	11.4	14	15	21
286	81	45	2.5	11.7	12.8	15.8	20
288	53	45	2.8	11	13.5	13.5	19
289	28	47	2.5	10.5	11.5	12.8	15
290	22	41	3	11.5	15.8	17	20
291	23	41	2.7	12	15	15.5	21

292	71	45	2.7	10	13	12.7	17
293	95	41	2.7	10.5	14	15.8	
294	76	47	2.9	10	14.8	15.8	20
295	35	47	3	10.5	13	16.1	22
296	105	41	2.7	12.5	15	16.2	20
297	20	42	3	12.2	15.6	17	22
298	82	47	1.3				
299	80	41	2.7	11.5	13.6	13	17
300	4	47	2.5	10.6	12.9	13.8	18
301	91	42	2.6	12	15.3	15.8	17
302	89	41	2.8	10	13	13.7	20
303	8	42	2.8	12	16.5	16	21
304	6	45	2.6	13	16	17.5	23
305	79	47	2.5	9.4	11.3	12	15
306	29	54	2.7	10.2	13.5	13.9	18
307	14	42	2.8	11.5	15	14.8	19
308	27	42	2.7	11	13.3	13.2	19
309	97	57	3.2	12	15	15.2	20
310	26	41	2.7	11	14.5	17	22
311	90	47	3	11.5	15	14.2	22
312	94	57	2.5	11.5	16.7	19.1	23
314	70	41	2.5	9.8	10.6	12	17
313	77	41	3	11.5	13.4	12.5	15
315	84	47	2.7	11.5	13	14	17
316	13	47	2.5	12	13.1	15.2	18
317	72	47	3	10.6	11.5	12.6	18
318	15	57	3	12.5	15.2	14	17
319	7	47	2.8	13	15.8	15.7	23
321	36	54	2.7	12	12.7	19	
320	50	54	2.8	12	13.8	15	20
322	16	57	2.6	11.5	13	13.2	16
323	92	42	2.6	10	12	14.2	21
324	96	47	2.6	10.5	13.5	15	18
325	74	57	3	12	15	17.5	22
326	68	54	2.8	10.5	11.5	12	16
327	65	57	2.7	10.5	13.1	14.5	17
328	30	42	3.6	12.5	12.9	14.2	20
329	110	47	2.7	11	12.4	13.2	15
330	31	45	3.2	13	15.7	16.4	23
332	85	42	2.6	12	13.2	12.6	17
333	21	47	2.8	12	13.3	14.2	16
334	100	41	2.7	11	11.5	13	18
335	59	41	3	10	11	11.9	
336	104	57	2.5	9.5	11.3	12.3	18
337	48	57	2.6	11	13.2	12.5	19
338	3	45	2.7	11	13.6	13.5	16
339	102	47	3	12			
340	86	54	2.6	11	13.5	18.1	
341	63	41	2.7	11.5			
342	10	42	2.9	11	12.5	16	19

343	2	45	3.4	13	14.5	17.7	21
345	12	47	3	12	14.5	18	
344	24	47	2.5	10	15.7	18.5	20
346	18	47	3.2	11	13.5	17.5	19
347	64	47	3	10.5	12.5	17.7	
349	69	47	2.6	7.2	12.8	14.2	14
350	9	47	2.9	8.5	11.5	16.6	
351	1	47	2.6	8.5	12.2	16.4	
352	56	41	2	7	10.2	12	
355	5	47	2.8	8	12.4	15.9	16
356	101	41	1.6				
358	73	41	2.6				
359	17	47	2.7				
360	62	41	3.3				
361	25	47	3				
362	11	47	1.5				
364	32	47	1.5				
365	38	41	2.6	10.5	15.9	16.9	18
366	81	45	2.6	8.5			
367	8	47	2.8	11.6	14.5	17.2	18
368	80	41	3	11.4			
369	72	45	2.5	11.2			
370	37	47	3	13			
372	48	45	2.5	9.7	15.8	17.8	18
371	71	45	2.4	8.4	15.8	18	19
373	28	45	2.5	11.8	15.8	18.4	19
374	50	47	2.4	10.9	15.9	19.3	19
376	23	41	2.7	13.1	18.6		
375	75	41	2.9	10.1	14.4	19.2	19
377	34	41	2.3	10.2	15.7	17.9	19
378	95	41	2.6	12.8	15.8	17	18
379	32	45	2.7	10.5			
380	20	47	2.8	11.6			
381	31	45	2	9.8	14.3	15.1	16
383	16	45	2.4	9.1			
382	97	45	2.3	9.4	14.5	17	18
384	77	41	3.5	11.9			
385	26	41	2.7	12.4			
387	14	47	2.5	9.6			
386	27	47	2.7	8.4	14.8	18	17
389	51	41	2.8	12.2	19.4		
388	52	41	3.6	12.9	19.5		
391	74	45	3.1	11.9			
390	94	45	2.4	11.9	18.4		
392	2	54	2.3	9.1	15.4	17.5	18
393	4	45	2.4	9.6	15.6	18.4	19
394	7	45	2.6	9.2			
395	13	45	2.4	10.2			
396	46	47	2.3	7.9	12.9	14.7	16
397	59	47	2.8	9.3	14.8	14.9	17

398	104	47	1.7	13			
399	85	41	2.3	9.1	13.4	16.5	17
400	102	54	2.2	10	14.1	16	17
401	89	41	2.8	11	15.1	17.4	19
402	92	47	2.5	9.2	14.7	16.8	17
403	43	41	3	13.1			
404	120	47	2.7	10.2			
405	24	54	3.1				
406	30	47	2.7	10.7	14.6	16.9	19
412	67	41	2.3	8.9			
411	254	41	2.6	9.1			
414	136	47	1				
413	238	47	2.1	6.8			
416	62	47	2.9	10.6			
418	66	41	2.9	9	13.2	15.6	19
419	273	47	2	9.6			
421	18	41	2.7	8.4			
422	103	41	2.4	6			
423	60	47	2.1	7			
426	235	41	1.9	6.5			
428	9	41	2.7	8			
430	21	47	2.6	7.6			
431	173	47	2.7	8.5			
433	5	41	2.8	7.4			
432	86	41	2.1	7.7			
435	73	41	2.2	8.1			
436	181	47	2.2	7.5			
437	11	41	2.7	6.4			
438	183	47	2.3	6.4			
443	56	41	1.5	5.7			
442	88	41	2.4	7.4			
448	1	41	2.9	8.4			
449	260	47	2.3	9.2			
450	267	47	1.9	7.4			
451	65	41	2.4	9			
455	44	41	2.6	6.1			
454	68	41	2.8	10.3			
459	88	314	2.4				
460	64	314	1.9				
462	73	272	2				
463	393	314	1.7				
464	67	314	2.1				
465	86	314	1.9				
469	420	314	2	5.2	12	13.5	15
470	439	314	2.5	8.8	15.5		
471	393	314	2	7.8	15.5		
472	434	314	2.1	13	13.6	13.8	
473	409	314	2	11.5	13.5	14	14
475	453	388	2.2	9.1	16.5		
474	457	388	2.2	5.8	12	13.2	14

476	446	388	2	7.8	15.5	17.5	23
477	458	388	2.2				
478	444	388	2.2	5	9.8		
479	441	388	2.2	5	10.8	12.2	15
481	447	388	2.3				
484	331	314	2	5.2	9		
485	429	314	1				
487	353	326	2.5	5	9.5		
489	185	45	2.3	8.2	15		
490	399	336	2	6.3	10.2		
493	251	222	2.3				
494	337	336	2	6.1	11		
496	374	57	2				
497	231	287	2.2	6.2	13.3		
501	270	326	1				
507	357	275	2				
508	415	326	2				
509	180	57	2.6	5	8.7		
510	371	271	2.1	6.2	11.7	15	21
511	276	287	2.7				
512	216	271	1.8	8.5	9.3		
513	332	222	1.3				
515	382	45	2.4	5.6	8		
514	189	45	1				
516	190	275	1.5				
517	121	275	2.1				
518	195	224	2.2	11.5	19.5	21.2	25
520	381	221	2.5	11.5	15.6	17.8	21
519	348	336	2.6	9.5	18	20.5	23
522	349	222	2.3	10.5	18.5	18	21
523	366	222	2	8	15.5	16.8	20
525	409	224	2.2	8.5	11.5	12.2	
524	420	224	2.1	6.8	12	12.8	17
521	191	412	2	9.5	14.5	21.5	25
526	401	222	2.2	11.5	19.5	19.8	24
531	439	222	2.2	11.6	18		
532	377	223	2.3	6	10.5	13.4	16
533	207	226	2.3	6.5	11.5	12.3	15
528	194	326	2.2	9	13.5	15.9	17
529	241	388	1.3	7	13.5		
530	269	412	2.3	7.8	11.8	13	16
537	399	223	2	7.8	13	14.8	20
539	410	225	2.2	7.5	12		
538	444	225	2.3	9.9	18.3		
534	201	326	2.3	9.5	17.5	18.6	22
535	206	336	1.7	7	14.5		
536	458	388	1.9	6.9	11.5	13	18
540	453	271	2.3	9.5	17		
541	306	326	2.3	8	13	14.6	16
542	406	388	2.1	8	15		

543	447	47	2.2	6	11.5	12.8	18
544	461	412	1.5	6	12.5	12.8	16
546	316	47	2.3	8	13	17.5	21
549	386	226	2.2	8	14		
548	390	271	2.1	8			
547	427	271	2.4	7.5	12	13.8	17
552	202	225	2.2	8.2	12	13.2	17
551	393	336	2.4	8	14	15.9	20
554	425	226	1.7	7.6	10.3	12	15
553	434	271	1.8	6	12.5	12	14
555	374	47	3	10.8	16.5	19	22
558	216	223	2	6.8	9.5	12	16
556	215	326	2.8	8.8	14	16.5	20
557	193	388	2.3	8.8	11.8	14.8	16
560	440	221	2	7	10		
561	352	224	2	7	10.5	11.1	14
559	276	336	2	6.5	10.5	12.2	15
563	212	47	2.1	8	13	13.8	15
564	214	221	2.2	8	13	16.8	17
566	199	225	1.9	5.8	10	12.2	16
565	197	412	2.9	10.8	15.5	16.8	21
569	302	226	1.7	7.5	13.5		
568	203	271	1.6	5.9	8		
570	210	223	2	6.2	12.5	13.8	15
571	333	224	1.4				
573	198	221	2.5	7.6	10	12.6	16
572	371	271	2.7	7	10.5	12.3	16
575	373	226	2.6	7.8	12	12.9	15
576	355	222	2.2				
577	354	225	2	7	11.6	13.5	16
578	407	47	2.3	9	13	13.8	16
579	396	222	2	6.4	8.5	13.4	17
580	415	47	2.2				
581	424	225	2	6.2	10.5	11.4	16
583	218	222	2.3	6.8	10.5	13.5	17
582	417	336	2	6	9	11.2	15
584	441	222	2.1	8.2			
585	208	222	1.5	5.2			
586	211	222	1.7	6.2			
587	220	222	1.3	5			
588	408	222	1.6	5.6			
589	329	222	1.5	6			
590	457	222	1	5.3			
591	400	222	1.4	6			
592	467	222	1	5			
593	212	222	1	6.2			
594	209	222	1.4	5.4			
595	213	483	3.1	11.8			
596	420	336	2.5	7.5	10.8	13.5	19
597	452	225	2.4	7			

599	415	226	2.5	8.6	12	15.5	17
598	406	336	2	7.5			
600	201	47	2				
601	371	224	2.2				
602	204	225	2.3	5.8	8.5	11.5	16
603	316	483	1.7	8			
604	333	226	1.8	7.2	12.5	15.8	21
605	429	226	2.5	8.5	13.1	15.5	23
606	482	483	2.5	7			
607	202	47	1.6	7.9	12.5	15.2	20
608	189	224	1.8	10			
609	191	483	2	9			
611	199	225	2.1	6.2	9.5	13.5	14
612	349	226	2.7	6.8	12	15	16
610	472	336	1				
613	320	224	2.3	9.2	15	17.4	21
614	441	47	2				
616	218	224	2.1	6.5			
615	192	336	2.4				
617	390	226	2.9	10			
618	445	226	2.6	8			
620	417	226	3	9.4	11.5	12.5	16
621	458	226	2.6	8			
622	241	226	2.5				
623	208	224	2.1	9	13	18.5	19
624	444	483	1.9				
626	210	224	2.8	6.9			
625	216	224	2.6	9.2			
627	407	225	2.6				
628	193	47	2.7	6.9	14	20	21
631	348	225	2.6	6.5			
630	480	225	2	6.2	10.5	16	17
629	381	336	2.3	6	13	17	18
632	306	483	2				
633	447	336	2.2	8.6	11.5	14.5	18
635	401	336	3				
636	209	47	1.7	6.5	11	16.5	17
637	410	224	2.2	6.5	12.5	17	18
638	363	47	1.9				
639	220	224	2.8	6.7	12.5	16	19
640	468	225	2.2				
641	473	225	2.1				
642	408	225	2.7	5.8	10	14	17
644	424	224	2.8	7.5	12.5	15	19
643	197	224	2.3	6	12	12	14
645	215	226	2.9	7.8			
646	399	226	2.7	6.8	10	13.5	18
647	196	47	2.5	7.2	11	15	16
648	205	47	2.4	7	10	13.8	15
649	409	483	2.2				

650	461	225	2.3	7	9.5	12	14
651	466	224	2.3				
652	302	483	2.5				
654	207	224	2.6	9.5			
655	480	483	2	6.8			
658	377	495	2.8	7.2			
660	329	224	2	8.6			
662	333	495	1.8				
666	492	225	2	7.8	8.8		
671	355	225	1.2				
673	400	495	2.8	8	8.7		
672	209	495	2.2	5			
674	445	225	2.5				
677	348	483	2.2	7	9.2		
679	468	495	2.4				
680	444	225	1.8				
681	474	483	1				
682	198	495	2.1				
683	408	483	2.4	5.3			
684	551	483	2.3				
685	407	495	2				
686	456	224	1.6				
687	473	224	2.3				
689	500	495	2	5.8			
690	543	224	2	5			
691	204	224	3				
692	461	225	2	5.1			
693	213	495	2.8	6.7			
694	386	225	2.2				
695	320	495	2.5				
696	366	225	2.3				
697	327	224	2.2				
699	624	225	1.7				
700	630	483	1.7				
702	468	225	2	9.3			
703	452	510	2.4	13.5	20.5	22.5	25
704	219	495	2	7.8	12	12.5	15
706	410	495	2.5	6.8	9	14	
705	533	495	2	7.6	10	12.8	
709	563	224	2	7.6	10.6	14	17
711	401	225	2.5				
710	589	225	2.5	13.6	14.5	18.5	21
712	458	495	1.5	6.4	10.5	12.5	
713	524	510	2	6.8	10.5	12	15
714	545	526	2.5	8.2	9.5	13	17
715	200	224	2				
716	409	483	2.5	6.1	8.5	12.5	15
717	407	495	2.5	6.2	11.5	14	18
718	532	510	2	9.7	10		
719	473	225	2.5	6.4	10.5	12.5	16

720	191	225	2.5	9.6	12	14	18
721	582	526	2.5	7.9	10.5	12	
724	554	510	2.5	7.6	9.5	13	16
725	217	495	2.5				
727	474	224	1.5				
726	565	224	2.5	6.5	8	11	
728	208	495	2	8	10.5	14	17
730	535	483	2				
731	213	510	2	7.1			
732	204	526	2	8.8	10.5	14	19
736	386	510	2.5	6	10	11.5	
735	417	510	2.5	7.5	9.5	14	17
738	567	225	2				
740	655	510	1.5	5.8			
741	196	483	1.5	5.1			
743	562	224	2	6.1			
744	349	225	1.5	5.6			
745	492	510	2	6.2			
747	500	224	2	6.4			
757	620	495	2.1				
758	636	495	2.5				
759	524	756	2.6	10.3	15.3	16	18
760	653	706	2	8.5	14.3	15.8	
761	582	639	2	8.2	12	15	
762	667	495	2.9	8.5	14	16	19
763	409	755	2.4	10.6	15.8		
764	218	495	2.1	7.5	12	13.5	15
765	535	526	2.7	9.6	13.8		
766	555	706	3	12.8	15.6	17.6	19
767	554	495	2	9.4	11.1	12.6	16
768	663	526	2	6.8			
769	545	635	2	7.2	10.5	13.8	19
770	611	706	2.5	11.2	15.3	17.5	20
771	558	635	2.5	6.4	8.5		
772	377	755	2.5	9.5	13.5	15	
773	492	756	3	13.2	18	20.6	23
774	217	635	2.5	9.4	13.6	15	
775	196	495	2.5	9.8	11.9		
776	589	635	2.6	10.5	15	20	
777	469	639	2.5	8.5	12.2	13.6	
778	550	706	2.5	9.4	12	13.5	17
779	482	706	2	8	11.8		
780	468	756	1.5	8.8	12.8	13.8	16
781	473	639	2	7.8	11.7	13.5	17
782	417	495	1.8	6			
783	665	756	2	7			
784	213	756	2.8	9.1			
785	619	756	2	7.8	10.8	12	
786	386	495	2.2	7.7			
787	527	526	2.2	6.8	8.5		

788	567	639	2.6	7.2	8.5			
789	424	755	2	5.8				
790	532	495	2	8.7	9.2	12		
791	678	495	2.4					
792	698	495	2	8.2	11	13	16	
793	401	526	2.5					
794	634	711	3					
795	665	711	2.5	11	14.7	18	20	
796	530	755	2.5	11.8	15.5	22.5	24	
797	468	713	3	12	18	25	27	
798	555	755	3.5	13.1	17.5	22.5	27	
799	656	666	2.5	10.5	13	15.8	18	
800	482	755	2.5	11.6	14	20	21	
801	750	702	2.5	10	12.3	15.5	17	
802	554	755	2.5	11				
803	733	754	2.5	8.5	11.5	16.5		
804	675	639	2.5	9.3	11.8	13.5	16	
805	748	711	2	9.8	11.9	15.8	15	
806	670	755	2					
808	474	702	3	9.5	11.5	14.4	15	
807	500	702	2	8.6				
810	657	666	2	5.2	8.8	13	15	
811	664	702	2	7.5	10.6	12.6	14	
812	473	754	2.5					
813	726	754	2	6.8	11	13.8	15	
815	545	702	1.5	7.2	10.3	16.2		
816	669	702	2	8	10.1	13.4		
814	710	702	2.5	10.5	12.1	15	16	
817	570	713	3.5					
818	668	755	1.5					
820	663	711	3	11	13	15.4	16	
821	735	753	2.5	11.5	13.8	16	16	
822	553	713	2	7.5	10.2	12.7	14	
823	722	753	1.5	6.8				
825	611	713	3	9.5	11.5	17	19	
824	708	713	2	6.8	9.5			
826	729	753	2	6.5				
827	724	754	1.8	6.8	8.3	11.5		
828	720	639	3	8.2	9.8	13.5		
829	582	711	3	7.8	9	11.5		
830	723	711	1.8	5.2				
831	667	702	2	7.8	10.2	13.5		
832	749	753	2.5	7	8.6	13	14	
833	688	666	2	7.5	9.4	14	16	
834	730	639	2	6.8				
835	637	711	3.5	11.5	13	16.8	19	
836	676	639	2	7	8.6			
837	213	711	2.5	9.8	11.3	15.8	18	
838	409	753	2	6.4	8.9			
841	570	753	2	6.6	12.5	16.5	21	

842	558	754	2	6.1	10.5	14	17
843	619	755	2.5	6.3	9.8	13	20
844	665	711	2.5	9.4	16.5	19.5	22
845	675	711	2.5	7.8	11.6	14.5	18
846	708	756	2	7.2	14.1		
848	734	633	2.5	6.7	14	19.5	22
850	554	711	2.5	6.6	9.1		
849	663	713	3	7.4	14	18.5	22
851	634	756	2	5.7	13.1	18	19
852	656	702	2.5	8.6	15	16.5	21
853	670	753	2	8.2	12.9	16	17
855	611	754	3	8.4	13.5	17.5	18
854	737	754	2				
856	468	755	2	7.1	11.9	14.5	17
857	751	756	2	5	12	16	17
859	553	711	1				
858	661	713	2.5	6	11.6	15	18
860	742	753	2.5	6.5	14	18.5	20
861	735	702	2.8	10.8	18.5	21	23
862	730	754	2	7	13	17	20
863	688	756	2.9				
865	377	633	2.3	8.7	13.6	16	18
866	716	711	2.3	6.9	10.5	12.5	15
867	749	753	3.1	7.9	11.8	17.8	20
868	676	754	2	7.5	16	21	24
869	739	755	2.5	6.5	11.6	14	16
870	669	702	2.7	6.6	12.5	16.5	18
871	701	756	2	6.9	11.4		
872	545	702	2.1	5.3	10.1	14.5	18
874	724	711	2.1	5.9	12	14.5	17
873	722	713	1.8	8.4	13	16.5	20
875	720	711	1.7	5.5	9	12	16
876	726	754	3.2	6	12	14.5	18
877	733	633	2.6	5.1	10	13.5	15
878	555	754	2.7				
879	409	713	2.4	6.7	12.5	18.5	
881	582	666	3				
882	678	666	2.9	9.5	15.5	22.2	21
883	752	666	1.4	5	10.5	15.5	17
884	746	756	2.1	5.5	13.4	13	17
885	723	754	2.4	6	11	14.5	17
889	806	702	2	8.5	14.2	16.7	19
888	820	702	2.5	8	12.2	12.2	17
890	789	702	3				
891	812	702	2	8.5	11	13.8	19
892	809	702	2.5	6.8			
893	723	756	2	8.3	13.4	14	
894	724	754	3.5	12.1	16.6	17.2	19
895	707	755	2	8.2	10.9	12.8	19
896	678	762	2.6	11.5	13.2	14.2	17

897	688	755	3.5	7.5	10.2	13.4	18
898	570	711	2.5	7.5	9	11	16
899	619	753	2.5	8	10.8	12.2	16
900	670	754	2.5	7.3	10.4	12.5	16
901	653	755	3	10.4	12.2	13.4	16
902	545	702	2.5	8.5	10.2	12.2	18
903	656	711	3	10.5	11.2	13.2	18
905	665	713	2.5	10.8	13	13.4	16
906	611	753	3	9.8	13.6	15.6	18
907	735	754	3	9	10.6	14.8	19
909	582	754	2.5	8.6			
911	742	754	3	10	13	12.6	17
912	659	762	3	8.2	12.5	15.6	20
913	722	702	2	9.4	10.5	14.6	19
914	492	702	3	9.1	11.5	12.5	17
915	524	702	3	8.8	13		
916	708	753	2.5	8.3	10.5	12.5	15
917	675	755	2.5	8.8	10.8	13.5	17
918	749	755	2.5	11.8	13.5	17	21
919	822	762	2.5	7.8	9.5	11.4	17
920	720	762	2.5	7.4	9.5	12.3	16
921	532	711	2	6.4	8.5		
922	377	756	2.2	7.4	11.6		
923	750	702	2.2	5.8	8.5	12.6	15
924	799	702	2	5	12.8	15.5	19
925	716	713	3	7.5	10		
926	845	713	2	7.5	10		
927	663	756	2	8.6	9	11.2	16
928	657	756	2.5	7.1			
934	678	860	2.5				
935	874	850	2.2				
937	661	865	2.2	7.5	11		
938	876	860	1				
939	663	762	2.9	8.9	12.8	13	15
940	818	762	2.2	9.5	13.8	13.4	15
941	735	753	2.2	6.7	10.2	12.8	
942	665	850	1				
944	722	825	2.6	10.8	12.2	12	14
945	708	860	1				
946	856	868	2.8				
947	716	884	2.4	9.8	13	13.2	15
949	849	865	1				
950	840	868	2.5				
951	724	753	2.8	8.5	11.2	12.4	14
952	872	762	1.8	7.4	11.2	13	15
954	861	753	2.6	6.5	8.6	11.8	
955	656	762	1.8	8.3	11.4	14	14
956	730	753	2.8	8	10.4	12.4	
957	742	753	2.2	7.1	8.4		
960	574	762	2.8	7.8	11.2	14.2	17

961	819	865	2.2	7.8	11.6	14	14
962	750	762	2.4	6.9			
964	855	753	2.4	7.8	9.8	11	
966	883	762	2.8	6.8	10.4	12.2	
967	839	865	1.9	6.3			
968	739	762	2	7	10.6	12.6	14
969	842	825	1.8	9.6	10		
972	759	762	2	8.6	9.2		
975	739	762	2.4	7.4	9	13.8	16
978	723	860	1.9				
979	656	762	2.2	8.8	11.6	17.4	19
980	858	860	2.2	6	9.2	13.2	17
981	872	868	2.2	6	8.2		
984	841	868	1.9	11	16	19	22
986	874	762	2.4	8.2	9.6		
988	750	860	2.2	7	8.8	13.6	
989	832	868	2.4	9.2	12.2	18	
991	676	868	1.9	5			
992	751	868	2	5.6	8.8	12.8	15
994	866	868	2.4	6.2			
995	869	762	2.4				
996	737	860	2.2	7.2	10.6		
997	716	825	2.6	5			
998	847	825	2	6.2			
1000	819	913	1.3	6.1	14		
1001	861	887	1.8	11	13.8	16.8	17
1003	724	868	2	6.4	11.8	14.2	16
1005	789	860	1.4	5.8	9.2		
1006	894	887	2	6			
1017	910	887	1.4	6.7	8.8	13.2	15
1018	899	887	2.2	10	13.2	18	18
1019	908	887	1.6	5.8	11	15.4	17
1020	912	911	2.2	6.6	9.8	13.4	14
1021	853	860	2.4	7	11	14	14
1022	927	911	2				
1023	842	867	2.4	8.2	10.8		
1024	872	860	2.2	9	10.8	15.5	18
1025	794	867	2.4	8	11	16.5	19
1026	866	860	2.4	7.8	11	17	20
1027	849	867	2.2	6.8			
1028	874	860	2	7.6	8.8		
1029	759	867	2.8	6.4	9		
1030	858	913	2.6	7	9.4	16	17
1031	939	867	2				
1032	955	860	2	8.2	12.4	15	
1033	708	867	2.6	8	12	13.5	
1034	722	867	2.8	8	9.4	15.5	19
1035	822	911	2.2	7.2	9		
1036	886	860	2.8	6.2	9.8		
1038	933	918	2.6	6.2	9.5	13	15

1039	861	918	2	6.8	12.2	15.2	19
1040	899	913	2.4	8	12.5		
1042	908	867	2.2	7.2	11.1	14	16
1043	864	918	2.2	7.4	8.4		
1044	869	867	2.2	8.6			
1045	963	860	1.8	9.1			
1046	953	867	1				
1047	968	860	2.4	9.4	13.6	16.4	20
1048	965	913	2.2	8	9		
1049	809	918	1.8	8	10.2	12.6	16
1050	880	918	1.5	6	9.5	13	15
1051	841	860	2.4	8.3	10.5	12.6	15
1052	839	868	2	6.7			
1053	888	867	2.6	7.4			
1055	914	860	2.6	7.2	11.5		
1054	919	860	2	7.5	8.8		
1056	875	918	2	7	9	12	15
1057	904	913	2	5.9			
1058	936	918	2.2				
1063	874	930	2.8	11.2	13.2	20	23
1064	955	973	2.3	11.2			
1065	980	860	2.4	10.8			
1066	872	867	2.6	9.2	12.8	19.6	24
1067	983	913	2	8.4	9.2	12	16
1068	866	930	2.6	9	11.2	17.2	20
1069	894	913	3	8.8	10.4	13.2	17
1070	873	913	2.6	9.8			
1071	993	973	1.6	8	9.4	15.2	18
1072	939	974	2.2	12.2	16.8	20	22
1073	937	974	2.8	7.6	8.6		
1074	999	974	2.5	10	15.9	20	21
1075	923	860	2.8	10	14.6	20.4	24
1076	990	867	1.5				
1077	849	913	2.6	8			
1082	841	970	3	10.2	13	14	
1083	888	973	3	10.4	15	18	22
1084	899	974	2.6	13.4	21.6	25	
1086	809	973	3	7.8			
1087	1001	974	1.8	7.2	13.2	16	
1088	864	970	2.4	8	11	13.4	
1089	948	973	2.2	5.6	8.2	11	
1091	818	974	2	8.2	11	14.4	
1093	891	974	2.4	10.2	18	20	
1092	977	974	2	7.6	9	12	14
1094	976	1014	2	7.8	13.4	16	
1090	982	1059	2	5.4	8	12	14
1095	875	1014	2.8	9.6	11.4	15	17
1097	886	973	2.4	5.8			
1101	912	973	2.2	7.4			
1102	908	974	2.6	7.8	10	14	17

1099	958	1059	2.4				
1100	963	1059	2.2	6.4	9.6	14.2	
1098	1002	1059	1.8	5.6			
1104	933	970	3	10			
1103	944	1059	2.6	7.6	12	15	
1106	916	1014	2.6	7	10	14.4	15
1105	968	1059	2.6	7.8	11.8	16	18
1108	919	1014	2.8	10	11	15	16
1109	943	1014	2.4	7.2	12	13	
1107	953	1059	2.3	6	9.2	13	15
1110	936	973	2.6	9	12	15.4	
1111	932	973	2.4	5.4			
1112	832	1014	2	7.5	14	16.6	
1113	914	974	2.2	6.2	10.6	13.2	16
1134	873	1015	2.6	11.2	15	19.1	20
1135	939	973	2	10.2	15	18	19
1140	990	1015	2.4	11.2	18	20	21
1139	980	1061	2.4	11	17.6		
1141	872	973	2.6	10.2	17.4	20	20
1142	894	974	2.2	11.4	14.2	18	18
1144	920	973	2.4	8	14	17.6	
1143	874	1060	2.2	11.4	16.6	17.6	19
1145	866	1015	2.8	9.2	14	18	18
1146	794	974	3	10.4	17		
1147	955	1014	2.4	8.8	13	15.6	17
1150	999	1015	2.8	9	11.2	16	
1151	1028	974	2.2	7.2	12.8	17	
1153	1049	973	2	9.4	11.6	17	
1157	977	1015	2.9	9.4	16	20	
1156	908	1060	2.8	8	11.4		
1158	910	1059	2	6	10.4	14	
1161	809	1060	2.8	8.8	14.6	19	
1163	927	1014	2	8	10.4	14	16
1164	869	1015	2.6	7.4	11	15	
1165	968	1016	2.8	8	13	15.5	15
1162	912	1060	2.8	8.2	11.2	14	17
1166	916	1059	1.9	7.2	13.4	16	
1168	965	1016	2.4	7.4	12.6	14	
1170	985	1016	2.4				
1171	944	1016	2.4	9	11.8	15	16
1172	1004	1015	2	7.8	14.8	17	
1173	853	1014	2.3	7	11.2	13	14
1174	914	1059	2.4	6.2	9.2	13	16
1175	1001	1060	2.2	7	12	16	19
1176	959	1060	2.4	7	12	16	19
1177	976	1015	2	9	14.5	15	19
1178	975	1059	2.4	9	14.2	19	21
1179	899	1014	2.6	10	13.2	16	19
1180	936	1059	2.6	8.2	12	17	21
1181	896	1014	2.6	10	13	15	19

1184	987	1016	2.6	9	11	13	20
1185	1037	1060	1.5	8	10	12	17
1186	932	1060	2.8	8.4			
1187	955	1152	2.4	11.4	19.6	22	
1188	939	1152	2.2	9	13	15	19
1190	866	1062	2.6	8.6	16.4	18	22
1191	1025	1152	2.4	8.6	12.8	13	17
1192	1028	1152	1.8				
1193	965	1059	2.6	6.4			
1194	958	1075	2.2	7.4	11	16.2	16
1196	977	1062	1.8	6	12.4	15.4	
1195	1107	1062	1.8	6	9	12.8	14
1197	985	1059	2	8	15		
1198	968	1062	1.8	5	11.6	14	17
1199	948	1062	2	7.5	13		
1200	1004	1062	1.8	5.5	10		
1226	927	1060	2.8	10.4	15		
1227	983	1062	1.8	7.2	11.6	13.4	14
1229	888	1062	2.8	8	16	18.4	
1230	1042	1152	1.4	5.4	10	13	14
1232	975	1059	2.2	8	11		
1233	841	1075	2.2	5.4	11		
1264	1083	1059	2.4	6.2			
1290	965	1075	2.6	12.2	19.2	18.4	22
1291	1095	1075	2.4	9.4	12.8	13	14
1293	1028	1075	2.6	9.2	16.4	16	18
1292	1135	1075	2.2	11	17.4	19.8	20
1294	980	1075	2.8	10.4	18	19	22
1299	1085	1152	2.6	13	20.4	22	21
1300	955	1131	2.6	12.8	21.2	23	24
1301	1049	1152	3	13.2	21.6	21	21
1302	1141	1152	3.2	12.6	17.6	22	22
1303	958	1131	2.6				
1304	990	1075	1.6				
1308	866	1152	2.8	7	13.6	15.8	15
1321	977	1267	2.8				
1322	932	1269	2.8	9.4	16.4	17	18
1323	999	1096	2.6				
1324	1083	1289	3	10.2	16.2	17.2	19
1327	1090	1267	2	8.4	12	13.4	15
1328	1185	1267	1.9	8	14	15.8	
1331	1171	1269	2.6	9.8	17.2	20.8	23
1332	1147	1267	2.2	8.8	12		
1335	1105	1096	2.6	9	12.2	14.4	17
1337	1108	1096	1.8				
1342	1238	1265	1.8	6.5	8.8	12	14
1344	1106	1270	2.6	9.7	12.8	12.4	15
1343	1009	1270	1.2	8.6	12.8	15.6	
1345	1261	1269	2	11.3	17.2	20.2	24
1346	1247	1270	2.2				

1347	1135	1267	2.4	12.5	17.2	20.8	
1348	976	1267	2.6	12.1	16.4	23.6	22
1349	1007	1270	2.2	8.5	10.8	14.8	16
1350	896	1267	2	10.4	14.2	20.6	23
1351	1028	1265	2.8	7.9	15.2	21.8	
1352	980	1267	2	8.5	13.8	20.4	
1353	1124	1270	2.4	9.7	15.6	20.8	21
1354	968	1267	2				
1355	1012	1268	1.8	5.4	9.8	14.8	15
1356	971	1265	1.4	7			
1357	931	1267	1.8	7.6			
1358	1176	1131	2	11.2	17.2	21.6	
1360	1182	1263	1.8	12.2	19.2		
1359	955	1267	1.6	7.8	12.6	17	20
1361	1165	1267	2.8	11.4	14.8	14.2	20
1363	1128	1270	2.8	13			
1362	1246	1270	2.2	9	15.8	20.6	20
1365	1113	1131	1.8	9.2	14.8		
1364	1013	1265	2.2	11.4			
1366	1275	1267	2.8	13	16.9	24.1	22
1368	1129	1267	1.2				
1369	1188	1267	2	5.8			
1370	929	1266	2.4	7	10.6	14.8	16
1371	1250	1267	1.6	6	10.2	11	15
1372	1278	1265	1.2				
1375	1010	1265	2	8.2	10.8	14.8	17
1378	1050	1265	2.3	9.8	14.4	21.2	24
1377	1173	1265	2.4	9.2	11.6	13.8	16
1376	1130	1265	1.6	7.4	12	17	20
1379	1155	1300	2.6	11.2	15	17.6	20
1381	1147	1265	2.2	10.4	14.4	16.6	19
1382	1271	1268	1.2				
1383	1257	1290	1.8	8.4	11.6	14.4	17
1386	1159	1300	2.2	9	10.6	13.4	16
1388	1154	1096	2.3	9.8	14.2	17.6	19
1389	1148	1270	2.4	8.4	11.8	15.6	19
1390	1137	1270	2.2	8.4	14.4	21.2	25
1391	1049	1290	1.4				
1396	1117	1224	2				
1394	1241	1290	2.2	5.8	8.8	12	17
1395	1256	1302	1.4	8.4	11.4	14.8	17
1398	1114	1300	2.2	5	10.4		
1400	1153	1265	2.2	8.8	13.8	19	25
1402	1244	1268	1.2				
1403	1244	1268	1.2				
1407	1126	1222	1.4				
1405	1067	1290	2.6				
1406	1286	1302	1.2	5.4	9	12.6	15
1409	1008	1270	2.4	7	10.2	14	16
1410	1115	1300	2.2	5.6	9.4	11.4	14

1414	1293	1224	2.8	5.4	9.8	12.8	15
1412	1193	1265	2.4	5.8	8	13	15
1413	1183	1290	1.8				
1417	1235	1290	2.4				
1418	1252	1300	1.8	6.8	11.8	18.6	23
1420	1242	1300	2.2	6.8	10.2	15.4	
1424	1122	1224	2.8	8.2	12.6	16.2	
1422	1125	1265	2	5.4	10.6		
1423	1243	1265	2.2	5.8			
1425	1195	1300	1.8				
1426	1198	1302	1.8				
1427	1011	1300	2.4	7.7	13.5	20.6	
1428	1138	1268	2.2	8.2	12.4	18	22
1429	1245	1270	3				
1430	1279	1300	2	6.8	10.4	13.2	14
1431	1042	1222	1.8	5	9.8		
1432	1282	1268	2.2	6.4	12.6	18.2	21
1433	1105	1290	2.2	5.2	8.6		
1434	1149	1268	2.2	5.2	10		
1435	1081	1270	2.6	5.8	10.2		
1436	1078	1270	2.8	7.2	12.2	19.2	23
1438	1047	1224	1.8				
1437	1259	1290	2.2	6.2	10	12.8	15
1439	1288	1224	1.6	6.8	12.2	18.2	21
1440	1251	1096	1.2				
1441	1239	1300	2.4				
1443	1178	1224	2.2	9.8	12.8	19	24
1445	1028	1223	2.4	8.8	12.2	16	17
1444	1176	1300	2.6	11.8	17.8	22.2	26
1448	1247	1300	2	7.4	10.2	13.4	16
1449	1119	1224	2	7.6	8.6	11	15
1450	1285	1300	1.8	7.6	10.2	16.8	16
1451	1287	1300	2.2	9.8	11.8	18.8	22
1453	1133	1290	2.2	7.2	9.2	13	17
1455	1254	1222	2.4	11.2	15.4	22	22
1454	1142	1300	2.2	8.6	10	14.6	16
1456	1128	1305	2.6	7	9.4	14	16
1457	1240	1305	2	7.8	10.2	13.6	16
1459	1248	1224	1.8	9	11.2	16.4	20
1458	1206	1290	2.4				
1461	1272	1225	2.6	6.2	10.8	15.4	18
1460	1235	1309	2.4	7.6	9.4	14.4	19
1463	1306	1300	2.2	10			
1466	1118	1305	2.2	5.4	8.4	13	15
1467	1214	1305	2.4	8.4	8.8	15.4	16
1468	1182	1290	2	8	10	14.6	17
1469	1208	1224	2				
1474	1155	1340	1.8	7.2	11.6	14.6	16
1475	1211	1330	1.6				
1476	1173	1330	2	6.2	10		

1477	1183	1340	2.4	5.2	10.6	14	16
1479	1171	1339	2.2	9.2	11.8	17.2	
1481	1198	1331	1.8	6.4	10.6		
1482	1101	1330	1.8	9	11	14.2	
1483	1185	1330	1.8	7.8	10.8	13.4	
1484	1324	1339	2				
1485	1319	1340	1.2				
1488	1105	1318	2	8	11.8		
1489	1311	1339	1.6	5.4	10	11.8	
1490	1237	1339	2				
1492	1207	1331	1.4				
1493	1297	1339	1.4				
1494	1245	1318	2.2				
1495	1165	1339	2.2				
1496	1217	1340	1.6				
1498	1201	1330	2	6.8	10	14	
1497	1122	1331	1.4				
1499	1212	1339	1.4				
1500	1153	1305	2	6.8	11		
1501	1215	1305	1.8				
1502	1271	1339	2				
1503	1259	1339	2	6.4	10.8	13.2	
1504	1132	1339	2.4	6.4	10.6		
1506	1251	1330	2.2				
1508	1114	1339	2				
1510	1148	1330	1.8				
1511	1284	1305	2	6	8.8	11.6	
1512	1129	1339	1.8	5.4	8.8	12.8	
1514	1338	1302	2.2	10.8	11.2	16.6	17
1515	1310	1302	1.6	11	11.4	16.4	17
1516	1188	1302	2.4	10.6	10.9	16.2	
1517	1261	1302	2	11	18	18.8	20
1518	1141	1320	2.2	12.6	18.7	25.1	27
1519	1178	1330	2.2	10.8			
1520	1182	1302	1.8	8	11.8	14.4	15
1522	1090	1302	2.4	8.8	15.6		
1523	1280	1331	2	10	14.8	16.6	18
1525	1285	1331	2.2	9	16		
1527	1307	1302	2.2	9.2	17		
1526	1253	1302	2.4	8	14	17	17
1528	1367	1330	1.8	7.4	14		
1529	1341	1340	1.8	6.6	13	15.6	17
1530	1259	1373	2.4	8	15.4	17.4	18
1531	1379	1330	1.8	6	10.6	15	17
1532	1249	1340	2.2	6.6	12	13.4	15
1533	1314	1373	2.4	6.8	11	15.8	18
1534	1195	1320	2.2	6.8	14	17	
1535	1257	1340	2.2				
1536	1127	1331	2.4				
1538	1248	1331	2.2	6	9.4	13.8	

1539	1255	1331	2.2	7.8	14.2	15.6	18
1542	1388	1330	2.8	9	16.4	18.8	18
1541	1101	1331	2.2	8	16.6		
1540	1220	1331	1.8	6.8	12	16	18
1544	1271	1340	2.8	10			
1545	1313	1340	1.8	5.4	10.2	11.8	
1543	1247	1340	2.4	6.8	12.4		
1546	1295	1373	2	7	13	14	16
1548	1298	1302	2.4	11.2	15.8	18	18
1551	1149	1330	2.2	12	16.4	19	21
1549	1133	1331	2.2	8.2	13	16	15
1550	1204	1331	2.2	6.2			
1553	1153	1302	2.2	12	17	21.2	23
1554	1375	1330	2	11.6	19	22	23
1555	1129	1340	2.2	9.4	13.2	16.2	16
1557	1254	1330	2.2	10.2	17.2		
1556	1370	1331	2	7.8	12.8		
1559	1177	1373	2.8	12.8	20.2	23.4	23
1558	1213	1373	2	10.6	17		
1560	1183	1373	2.4	8.8	13	17.8	17
1561	1121	1302	1.8	9	11.4	15	14
1562	1113	1330	1.8	6.2	10		
1563	1236	1330	2	9.6	16.6	20.4	19
1564	1203	1340	2.2	9.4	12.4	16.4	16
1565	1209	1373	2.2	10.8	14.8	19.2	19
1566	1262	1302	2.8	13.2	18.2	21.2	21
1567	1306	1340	2.4	7.4	13.2		
1569	1329	1330	2.4	9.2	15.6	19	19
1570	1185	1348	2.4	12	16.2	19.2	18
1571	1361	1353	2.6	13.2	17.2	21	22
1576	1415	1320	2.4	8.2	14.8		
1575	1277	1320	1.8	7.8	13.8		
1574	1237	1331	3	8	14.2		
1573	1245	1331	2.8	12.8	18.4	20.2	
1572	1205	1331	2	6.8	12.2	17	
1578	1228	1340	2	8.8	14.2	19.6	19
1577	1317	1340	2	6	10.4		
1579	1316	1373	1.8	8.6	15.2		
1580	1251	1302	2.8	8.8	15.6	19.8	
1581	1315	1320	2.8	13	15.4	17	16
1582	1122	1340	2.4	7.8	13	18.4	17
1584	1312	1340	2.4	7.4	10.2		
1585	1230	1330	2.6	8.4	14.4	19.2	21
1587	1041	1373	2	9.2	14.8		
1588	1278	1373	2.2	8.2	12	16.2	16
1586	1167	1391	2.4	8.8	16		
1589	1216	1331	2	6.8	10.8		
1590	1067	1394	2.4	7.8	11.2	16.8	18
1591	1137	1302	1.8	5.2	9.6	13.4	
1592	1123	1340	2.8	10.4	12.8	17.2	

1593	1136	1339	1.8	8.6	13.6	17.2	18
1594	1283	1428	2.2	8.4	13.4	17.4	16
1595	1094	1302	2.6	6.8	12.2	17.8	18
1596	1319	1391	2.4	9.8	13.4	19	18
1597	1279	1394	1.6	7	14		
1598	1381	1439	2.4	9.8	14.8		
1599	1260	1433	2.6	8.2	13.2	17.4	17
1600	1179	1436	2.2	8.6	11.8	15.2	
1601	1120	1391	1.8	7.8	14.4		
1602	1386	1400	2.4	9.8	14.2	17.6	19
1603	1211	1418	2	9.8	16		
1604	1080	1439	2	7	10.8		
1605	1160	1433	2.4	8.6	13.2	16.8	16
1606	1399	1320	2.2	9.8	15.8	19.4	21
1607	1296	1418	1.8	7.4	10.8		
1608	1274	1436	2.4	10.4	13	17.4	17
1609	1215	1302	2.2	9.2	12	15.4	14
1610	1275	1418	2.4	10.4	15	20.4	19
1611	1081	1394	2.2	7	10.2		
1612	1128	1433	2	5.2	9		
1613	1234	1320	2.6	7.8	13.2	17.8	17
1614	1154	1391	2.2	5	10.4	13.4	14
1615	1311	1433	2.2	8.8	12.2	15.4	14
1616	1252	1331	2	8	11.4	14	14
1617	1437	1409	2.2	6.6	11	13.6	14
1618	1079	1302	2.2	7	10.8	14	15
1619	1258	1421	2.2	10.8	16	21	20
1620	1325	1439	2.4	6.2	11.4	14.8	15
1621	1240	1302	2.4	7	10.2		
1622	1125	1436	2.4	8.2	11.6		
1623	1135	1302	2	6	10.6		
1624	1244	1340	2.2	9	13.4		
1625	1355	1436	2.8	8	12.2	16.2	
1626	1312	1331	1.6	6.4	9.2		
1627	1392	1373	1.6	5.6	9.2		
1629	1349	1330	1.6	10	12.2	15	16
1630	1148	1339	2	8	8.4		
1631	1335	1302	2.6	11	16	19	25
1632	1256	1340	2.2	9	13.2	16	19
1633	1198	1348	2.2	9	10.8		
1634	1416	1302	1.6	9.4	13.8		
1635	1410	1320	2	8	11		
1636	1288	1330	2.8	12.4	17	20	24
1637	1235	1330	2.6	12	13.8	16	16
1638	1155	1302	2	7.2	9.8		
1639	1219	1373	1.8	5.4	11.6	12	14
1640	1105	1331	2.2	6.4	11.4	15	15
1642	1291	1340	2.4	6.8	9.2		
1643	1360	1331	1.8	5.4	10.8		
1644	1171	1330	2.8	6.6	10		

1691	1188	1400	2.8	11	15	19	25
1694	1132	1373	1.8	8.8	12.4	11.8	18
1692	1343	1401	1.6	10.4	12.6	14	18
1693	1159	1433	2	10			
1695	1214	1394	2.4	13	15.2	15.8	19
1696	1241	1400	2.4	6.8			
1697	1116	1400	2.4	11	14	15	20
1698	1138	1373	2	8.2	12	12.2	16
1699	1282	1433	2.4	10.8	13.2	16.4	21
1700	1231	1439	2	12.2	15.2	17	21
1701	1206	1401	2.2	9			
1702	1297	1433	2.8	11.4			
1703	1326	1433	2.6	11	12.4	12.6	18
1704	1322	1400	2	10.2	14.2	16.4	20
1705	1273	1400	2.4	12			
1707	1430	1439	2.4	13.4	19	21	23
1706	1202	1439	2.6	13	15.2	15	19
1708	1313	1401	2.4	12.6	15.4	16.2	20
1709	1218	1433	2.8	12.8	14.6	15.2	17
1710	1284	1373	2.2	9.8			
1711	1336	1401	2.4	10.6	14	15.6	18
1712	1310	1394	2	10.2			
1713	1174	1433	2.2	10.8			
1714	1142	1433	2.6	12.5	16	19.7	27
1715	1406	1433	2	9.4	10.8		
1716	1182	1433	2.6	11			
1717	1287	1433	2.4	11.2			
1718	1333	1439	3	12.8	15	16.2	18
1720	1195	1394	2.4	13	19		
1719	1115	1394	2.6	11			
1721	1090	1400	2.8	12.2	14.2	16	19
1722	1169	1433	2.8	11.6	17		
1723	1285	1394	2.4	9.8	13	15	19
1724	1164	1439	2	7.8	10.2	12	16
1725	1380	1433	2.2	8.4	11.8		
1726	1271	1433	2.6	10			
1727	1234	1400	2.2	8.8			
1728	1258	1439	2.2	8.8	10.4	11.4	
1729	1243	1394	2.4	10.8			
1730	1307	1433	2	7	10.2		
1731	1442	1433	1.6				
1732	1136	1394	1.8	6	11.6		
1733	1167	1400	2.2	7	13.2		
1734	1395	1391	2				
1735	1198	1433	2.2	5	8		
1736	1399	1400	2.4	6.6			
1737	1208	1400	2.6	8.8			
1738	1334	1400	2.6	10.2	17.2		
1739	1278	1401	2.4	8	15	17	20
1740	1375	1391	1.6	5	10.2		

1741	1661	1394	2	10.2	15.8	19.2	20
1742	1312	1400	2	6.2			
1743	1251	1433	2				
1744	1274	1384	2	9	13	15.2	19
1745	1296	1391	2.2	6.6			
1746	1335	1409	2.4	8	13	15.2	16
1747	1377	1384	2.2	11.2	16.2	18.2	19
1748	1338	1433	2.2	7.8	13	16.8	19
1749	1254	1439	2.6	8.8	13.4	16.2	17
1750	1379	1394	1.8	7	10.4	13	
1751	1686	1439	1.8	7	13	18.2	
1752	1360	1384	2.2	8	12.2	15.6	18
1753	1203	1418	2.6	11.6	19.4	24.2	27
1754	1462	1428	2.2	9.4	14.2	18	19
1755	1374	1384	2	8	14.8	21.4	24
1756	1204	1391	2	7.4	12.8	16	18
1757	1473	1418	1.8	5.8			
1758	1212	1418	2.2	7.2	12.2	15	16
1759	1387	1436	2.2	9	14.2	17	22
1760	1220	1400	2.4	8	13.6	15.8	18
1761	1275	1428	3	7.8	13.2	16.2	19
1762	1249	1436	1.8				
1763	1183	1391	3	10.2	13.2	16.2	19
1764	1215	1391	2.6	7.2	9.2	12	
1766	1155	1428	2.2	7.4	11.6	15	18
1765	1252	1428	2	5	8.2		
1767	1671	1436	2.8	10	16.4	19.8	21
1769	1361	1418	2.8	8.2	12.8	16	22
1768	1651	1418	1.8	6.8	12	15.8	17
1770	1680	1428	1.8	7.2			
1771	1447	1433	2.4	7.8	14.2		
1777	1468	1373	2.4	8.8	14.4	17.2	19
1773	1383	1400	2.8	8.8	16.2	23	27
1774	1482	1400	1.4	5.2	11.2		
1775	1276	1436	2.6	7.2	15.2	19	
1776	1281	1439	2.2	5	9.2	13.8	21
1778	1171	1439	2.2	5.2			
1779	1407	1439	2.2	9.8			
1780	1205	1428	2	10	14.8	18	19
1781	1160	1436	1.8	7.4	15.8	17	23
1782	1674	1400	2.4	8.8	14.2	18	
1783	1464	1418	2	7.2	14.2	18	21
1784	1672	1391	1.8	7.4	13.8	16.2	18
1785	1654	1436	2	5.8	9.6		
1786	1376	1394	2.4				
1787	1341	1436	2.2	9	13	15	17
1788	1677	1436	2.4	9.2	15.2	19.6	25
1789	1659	1394	2	10.8			
1791	1676	1439	2	5.4			
1790	1216	1439	2.4	10.2	15.4	18.2	22

1792	1349	1436	1.8	5.2	10.4		
1793	1314	1436	2	8	12.2		
1794	1509	1436	2	8.4	15.2	20	
1795	1662	1436	1.6	6.2			
1796	1470	1409	1.6	6			
1797	1455	1428	1.8				
1798	1648	1391	1.8	5	9.4		
1799	1325	1428	2.4	8	14.4	19.2	23
1800	1666	1428	1.8	6.4	11.8	15.6	18
1801	1367	1391	2	8	10.4	13.2	
1802	1311	1436	2	7.8			
1803	1411	1436	2.6	8.2	16	20.8	27
1804	1653	1384	1.8	6.2	9.2	12.2	
1805	1213	1418	1.6	6.6			
1806	1207	1436	2	6.2	11	15	
1807	1645	1439	1.8	7.6			
1808	1329	1391	2	7			
1809	1319	1400	2.2	9.2	15.4	20.2	23
1810	1478	1401	2.6	8.8	12.6	16.2	20
1811	1386	1409	2.6	6.2			
1812	1133	1436	1.8	6	11.6	15.8	19
1813	1450	1394	2.2	7			
1814	1480	1400	2.2	12	19.6	24.8	27
1815	1670	1418	2.2	8	13	15.2	17
1816	1154	1439	1.8	6.4	11.8	16.2	19
1817	1176	1439	1.6	7.6	12.2		
1818	1211	1394	2	7.8	14		
1819	1673	1409	2	6.4	8.2		
1820	1445	1394	2	7.2			
1822	1471	1384	2	6.6			
1823	1397	1428	2	6.8			
1824	1221	1384	2.4	9.2	17.2	20.8	
1826	1675	1436	2	7	10.4	15	17
1825	1242	1436	2.6				
1827	1505	1391	1.6	6			
1828	1189	1428	2.2	10.2	17.2	23	27
1829	1437	1384	2.4	6.8			
1830	1306	1400	2.4	9	16.2	21.2	27
1831	1669	1439	1.8	6.2			
1832	1279	1436	2	7.2	11		
1833	1456	1400	2.8	7			
1834	1652	1391	1.6	6			
1835	1668	1400	2.8	8.4	16.2	22	
1836	1178	1428	2.2	7			
1841	1177	1446	2.4	10	11.2	11.4	16
1842	1483	1446	2.6	13.2			
1843	1551	1472	2.8	7.4	11.4	14.6	16
1844	1221	1391	2.6	11	16	19.9	25
1845	1153	1472	2.8	12	16.4	17.8	22
1846	1614	1373	2.8	11.2	17.3	22.2	27

1847	1406	1472	2	8	11.4	11.2	17
1848	1665	1513	2.2	9.6	13.7	17.2	20
1850	1271	1472	2.6	10			
1849	1287	1472	2	9	13.7	17	18
1851	1210	1513	3.2	11			
1852	1169	1472	3	12	16.6	20.4	
1853	1416	1472	2	10.2	12	15.4	14
1854	1448	1472	2.2	10.4	14.7	21	
1855	1452	1446	2.6	9			
1856	1217	1472	2.6	11.6	19.3	25	27
1857	1297	1472	2.4	8			
1858	1678	1513	2.2	8.2	16.1	19.9	27
1859	1547	1513	2.2	8	12.4		
1860	1343	1472	1.8	6.8			
1861	1684	1472	1.8	10.8	13.6	17.4	18
1862	1607	1513	1.6	6			
1863	1285	1513	2.2	10			
1864	1322	1446	3	13.2	16.6	23.8	26
1865	506	1513	3	10			
1866	1385	1513	2	7.2			
1867	502	1472	2.8	12	14		
1868	1532	1451	2	7.8			
1869	1389	1391	2.6	9.6	12.4		
1870	1465	1446	2.4	9.8	10.2	11.8	17
1871	1605	1513	2.2	13	14.6	15.8	19
1872	1552	1446	2	9.4			
1873	1595	1472	1.8	9	13	14.4	19
1874	1657	1391	2.4	9.4	13	16	16
1875	1296	1513	2.2	9.4			
1876	1564	1391	2.4	13.2	17	23.2	27
1877	1188	1446	2.4	8.2	12.4	18.8	21
1878	1377	1391	2	8.6	11.4	13.2	16
1879	1319	1472	3	9.8	10.4	14.8	
1880	503	1391	3	13.2	21		
1881	1419	1451	3.2	12.4	19	23	
1882	1682	1513	2	11	15		
1883	1376	1391	2.8	13.2	14.8	15.8	
1884	1383	1451	3.2	12.7	17.5	24.6	
1885	1455	1472	1.8	8	9.6	15	17
1886	1449	1513	2.2	8.2			
1887	1410	1472	2.2	10.4	14	15.8	18
1888	1278	1391	2.6	8.6			
1889	1392	1391	2	6.4			
1890	1203	1391	2.2	8.6			
1891	1470	1446	2.2	9.2			
1892	1663	1451	2	9.9	15.7	22	
1894	1482	1472	2	11.4	14	16.2	
1893	1315	1472	2.2	8			
1895	1392	1391	2.4	10.8	15.4	22	
1896	1149	1446	2.4	12.2	18.2	20.4	22

1897	1189	1513	2	11	15	18.2	20
1899	1341	1513	2.4	12			
1898	1468	1513	3	12.4	14.9	21.8	25
1900	1379	1446	2.4	7.4	12	17.6	18
1901	1375	1451	2.8	13.2	17.2	22.6	25
1902	1437	1472	2.6	9			
1903	1658	1391	2.8	9			
1904	1386	1451	1.4	8			
1905	1549	1513	1.6	9.6	14.8	21.5	
1906	1164	1391	3	7.4	9.6	12.6	19
1907	1509	1451	2	10.2			
1908	1198	1513	2.2	12			
1909	1462	1391	2.4	10.6	14	18	21
1910	1361	1446	2.2	9.6			
1911	1664	1391	1.8	8.8			
1912	1647	1391	2	8.8	11	15.4	20
1913	491	1446	1.8	7.8	14.4	17.6	19
1914	1480	1409	2.4	7.4	12.8	16.4	
1915	1628	1391	1.8	9.4			
1917	1404	1467	1.8	5	9.2	12	14
1916	1672	1467	1.8	8.6	15.6		
1918	1395	1472	2	6.8	11.4	14	19
1919	1608	1446	2.6	6.2	10.6	15	
1920	1583	1472	2	9.4	14		
1921	1637	1472	2	7	13.2	18.8	25
1922	1311	1467	2.2				
1923	1526	1472	2	6	11.4	13	16
1924	1671	1472	2.8	8	15.8		
1925	1531	1409	2.4	6.8	11.8	13.8	18
1926	1601	1446	2.2	7.4	13	15	18
1927	1668	1467	2	6.2	10.6	14	15
1928	1593	1472	2.6	7	10.6	13.6	18
1929	488	1513	2.8	7.6			
1930	1572	1467	2	5.8	11.8	17.4	
1931	1661	1513	3.2	9.4			
1932	1677	1391	2	8.8	11.4	14.2	17
1933	1675	1446	2.8	11.4			
1934	1594	1409	2.8	5.8	9	14.2	20
1935	1560	1467	2.2	5.6	11.2	13.6	15
1936	1486	1472	3	6.2	11		
1937	1518	1513	2.6	7	13.4	18	20
1938	1464	1537	2.2	7.6			
1939	1619	1537	2.8	6.8	10.6		
1940	499	1409	2.2	5.4	9.4	13.2	15
1942	1456	1446	3.2	9	16.4	19.6	21
1941	1615	1446	3	9.8	16.2	23.4	27
1943	1681	1517	2.6	8.4	11.8	13.4	16
1944	1399	1537	2.2	5.4	11	14.2	20
1945	1407	1391	2.4	6.8	11.6	15	19
1946	1690	1391	3	9.4	11.4	15.4	17

1947	1471	1472	2.2	5.6	11	15.8	21
1948	1666	1513	2.4	6.2	12	16.4	
1949	1450	1537	3	6.4	12.6	18	22
1950	1685	1409	2.8	7	11.8	15.4	17
1951	1497	1472	3	10.4	14	17	22
1952	1650	1472	2.8	11.2	15	19	25
1953	1654	1513	3				
1954	1360	1391	3	9.2	13.8	16.2	18
1955	1329	1472	3	9	13.8	18	
1956	504	1517	2.2	5.8			
1958	1662	1537	2.8	8	13.4	17	
1957	1683	1537	2.8	9.6	11.6	15.2	16
1959	1505	1391	3	9	12.8	15.8	16
1960	1408	1467	2.2	5.4	9.2	12.4	15
1961	1596	1517	2.6	8.4	14.4	17	20
1962	498	1391	2.6	8.8	14.2	21	24
1963	1670	1446	2	11			
1964	1370	1467	2	8.2	13.4		
1965	1515	1537	2.4	10			
1966	1646	1472	2.2	6	9	12.4	15
1967	1667	1537	2.4	7.8	15.2	19.8	21
1968	1387	1391	3	11.8			
1969	1645	1467	2	7	14		
1970	1648	1472	2	7.4	11.8	16.8	18
1971	1674	1513	2	6.4	11	14.4	15
1972	1568	1517	2.4	12	17.2	21.2	24
1973	1599	1517	2.2	9.7	15	17.2	19
1974	1652	1537	2.6	12	15	17.8	20
1975	1397	1636	2.4	10.2	14.4	15.4	18
1977	1656	1513	2.2	10.6	14	15	17
1978	1651	1557	2	7	10.2	14.8	17
1979	1367	1467	2.4	6	9.4	13	15
1980	1592	1537	2.2	8.8	14	19	21
1981	1514	1513	2.2	9.8	13.2	16.4	19
1982	1445	1517	2.4	8.8	13.4		
1983	1453	1557	2	9.8			
1984	1581	1587	2.2	9.8	14	16.4	20
1985	1673	1537	2				
1986	1349	1633	2.4	9.6	15.8	19.8	
1987	1521	1554	2.6	8.2	13	14.4	17
1988	1327	1554	2.2	6.2	10	13.2	15
1989	1580	1554	2.6	7	14	14.4	16
1990	1334	1636	2.2	8			
1991	1548	1467	2.4	5.4	11.8	14.4	16
1992	1616	1557	2	6.4	13	16.2	18
1993	1641	1636	2.2	7	11.6	16.4	18
1994	1520	1554	2.2	13			
1995	1231	1636	2	5	10.2	12.4	15
1996	1653	1467	2.4	7.5	11.4	16	17
1999	1511	1472	2	5	9.2	12.2	

2004	1523	1513	2.2	7	13.4	17.4	19
2006	1539	1557	2.2	6.4	14	18.2	20
2008	1555	1513	2.6	6.6	10	14	16
2009	1688	1517	2	6	12	17.4	19
2010	1564	1557	2.2	6	10.4		
2011	1699	1537	1.8	5.6			
2012	1393	1537	1.4				
2013	1442	1636	1.8				
2014	1487	1517	1.2				
2016	1659	1633	1.8	13.4	18	18.8	24
2017	1614	1633	2.2	13	17.4	20	27
2018	1437	1517	2	12.5	16.8	19.4	22
2019	1473	1524	2	11.6			
2020	1468	1537	1.8	10	14.4	18.2	22
2021	1530	1557	2.2	11.4			
2023	1771	1557	2	11.2			
2025	1410	1517	2.2	11	15	19.2	21
2026	1322	1557	2	13	16	20.4	27
2027	1686	1633	1.6	13.2	16.2	15	20
2029	1198	1537	2	11			
2028	1462	1537	2	13.2	16	18.6	24
2030	1753	1524	2	11.2	12	15	19
2031	1616	1554	1.8	10.4	12	13	15
2032	1703	1837	1.6	10	11.6	15.4	19
2033	1379	1557	2	12.8	16.8	20	24
2034	1607	1557	1.6	13	16	17.2	22
2035	1529	1517	2	10			
2036	1375	1837	1.8	13	15.4	17.4	21
2037	1640	1524	1.8	10.4	13.2	15.6	21
2038	1465	1537	1.8	11	12.8	14.4	18
2039	1665	1620	1.8	6.8			
2040	1271	1537	2	10.5			
2041	1380	1554	2	11.4	14	16.8	19
2042	1663	1513	2	12			
2043	1452	1517	2	11	16.4	19	22
2044	505	1554	1.8	11	15.6	19	23
2045	1680	1633	2.2	12	14.2	19.2	25
2046	1299	1537	2.2	13.2	16.2	19	23
2047	1617	1557	1.8	11	14.6	17	21
2048	1547	1620	2.2	11.4	13.6	17.4	22
2049	1655	1633	2	11.4			
2050	1386	1513	1.8	10	13	16.4	21
2051	1708	1620	1.8	13	14.4	18	
2052	1552	1557	1.8	10.8			
2053	1549	1837	2	12.4			
2054	1748	1524	1.8	9.8	10.4	12.6	17
2055	1821	1554	2	12.2	17.2	21	26
2056	1385	1517	1.6	9.4	11.6	15	19
2057	1704	1517	2	9.8			
2058	1546	1554	1.8	12.2	14.6	20.2	27

2059	1740	1837	2	10	11.6	12.8	18
2060	486	1524	2	11.4	14.2	16.8	23
2061	1509	1620	2	10	11.4	15.4	18
2062	1571	1633	2	8.8	13.4	17.8	25
2063	1411	1837	2	13	18.4	23.4	27
2065	1664	1537	2.2	9.1	11.8	15.2	19
2064	1709	1537	2	9.2	11.4	14	18
2066	1449	1554	1.8	7.8			
2067	1448	1524	1.8	10.8	12	16.4	21
2068	1682	1517	1.8	11.2	13.6	20	23
2069	1700	1537	1.8	11	11.6	14.8	22
2070	1684	1513	1.6	7	8.4	12	
2071	1376	1837	2	8	9.2	13	
2072	1419	1513	1.8	9	10	15	20
2073	1694	1517	1.8	10			
2074	1764	1557	2	11	14.8	19.2	21
2075	1361	1620	1.8	11	12.8		
2076	1470	1633	2	11.4	12.8	18.4	22
2077	1562	1524	2	8.8	10.2	16	20
2078	1624	1554	1.8	9.4	10.4	16.4	20
2079	1609	1517	1.8	11			
2080	1693	1517	2	8.8			
2081	1631	1554	1.8	10.4			
2082	1539	1513	1.8	10			
2083	1724	1537	2	8	10.6	16.4	19
2084	1526	1517	1.8	7.4	8.6	13	20
2085	1683	1537	2	10.4	11.6	17.6	20
2086	1532	1554	2.6	9			
2087	1656	1517	2.2	11.2	12	17	22
2088	1533	1554	1.8	8.8	9.8	15.4	20
2089	1657	1557	2	10.2	13.4	15.4	18
2090	1569	1524	2	6	8.1	11.6	15
2091	1699	1446	2.4	11	13.8	14.8	15
2092	1297	1446	2	8	16		
2093	1645	1446	2.6	10			
2094	506	1557	2	11	15.2	16.4	18
2095	1689	1817	2	9	14	15.6	18
2096	1759	1557	2.6	11			
2097	1531	1837	2	6	10.4	12.4	16
2098	504	1446	2	8.8			
2099	1568	1557	2	8.4	12.6	15.8	18
2100	1491	1633	2.4	11.6			
2101	1518	1817	2				
2102	1560	1837	2.2	9	16.2	17.8	19
2103	1453	1446	2	10			
2104	1688	1557	2.6	8.8	14.2	17	18
2105	1572	1620	2.4	10.2	16.2	17	18
2106	1747	1620	2.4	10.2	14.2	15.6	16
2107	1445	1636	2.2	9			
2109	1456	1446	2.8	10	12.2	15.4	

2110	1599	1557	2.4	8	13	15	16
2111	1647	1620	2.2				
2112	1641	1633	2.4	9.2	16.4	21	22
2113	1404	1557	2	7	13	14	16
2114	1806	1780	2.8	10	14.4	18.2	20
2115	1637	1817	2.2	8.8	12	15	18
2117	1514	1446	2.8	10.8	16.4	20	22
2116	1661	1446	2.8	9.8	13.4		
2118	1662	1557	2.2	10	15.2	17	18
2119	1430	1633	3	10	15.4	16	17
2120	1690	1636	2.8	10.8			
2121	1589	1840	2.6	9.9	18.4	19.2	21
2122	1695	1636	1.8	10			
2123	1583	1780	2	10.8			
2124	1540	1840	2	10.8			
2125	1723	1636	2.4	12			
2126	1450	1780	2	10	15.2	16	16
2127	1674	1446	2.4	9	15.4	17.8	19
2128	1676	1620	2	10	16	16.8	17
2129	1523	1780	2.2	10.2	12.2	15.8	16
2130	1581	1780	2.4				
2131	1608	1817	2.2				
2132	1507	1837	1.8	9	15.4	17	17
2133	1592	1633	2.2	11.4	18.2	17.6	19
2134	1756	1633	2.2				
2135	1652	1780	2.4	11	19.2	23.4	25
2136	1672	1817	2	9.2	14.2	16	17
2137	1675	1840	2.2	10	15	17.4	19
2139	1480	1446	2.2	9	13.6	15.8	17
2140	1670	1636	2	10.2	16	19.2	
2141	1752	1636	2.2				
2142	1658	1557	2				
2143	1666	1817	2	6.2	11.2	16.2	18
2144	1648	1620	3				
2145	1511	1817	2.2				
2146	1629	1636	2				
2147	1415	1772	2	9.4	12	16.4	20
2148	1367	1817	2				
2149	1781	1780	2	9.8	15.4		
2150	1639	1840	2				
2152	1660	1620	2.2	9.2	15.4	21	23
2151	1749	1620	1.8	8	8.6		
2153	1483	1636	1.8				
2154	1677	1446	2	9	13.2	17.2	20
2155	1668	1837	2.2	9.4	14	16.8	18
2156	1651	1446	2.8	10	14.2	18.2	24
2157	1610	1620	2.2				
2158	1383	1633	2				
2159	1667	1817	2.6	8.8	15.4	21.4	24
2160	1762	1840	2				

2161	1407	1557	2	5	12	14.8	17
2162	1739	1840	2	8	8.4		
2163	1548	1780	2.4	10	15.8	20.8	23
2164	1654	1446	2	11	18.9	23.6	24
2165	1377	1620	2.4				
2166	1530	1633	2.2	10.6	14	15.4	18
2167	1848	1772	2	12	16		
2168	1926	1780	2.2	11.6	15.4	16.4	19
2171	1628	1837	2.2	11	14	16.4	19
2170	1790	1837	1.6	8.8	12.2	15	
2172	1849	1817	2	8.4	10		
2173	1555	1620	2	10.6	12.2	14.8	19
2174	1464	1840	1.8	8	13		
2175	1673	1633	2.8	6	8		
2176	1844	1780	2	10.4	14.2	14.6	19
2177	1521	1636	2.4	10	17	19	20
2178	1681	1446	2.4	8.2	14		
2179	1887	1780	2	8.4	13	15.2	18
2180	1650	1633	2.8	11	16		
2181	1601	1837	2.4	11.6	19	19.8	23
2182	1442	1446	2.6	11	16		
2183	1580	1780	2.4	6.6			
2184	1763	1780	2.6	8.6	16	15.8	18
2185	1843	1817	2	5	10.2	14.2	16
2186	1862	1780	2.4	6.2			
2187	1706	1446	2.4	5.6			
2188	1615	1837	2	10.2			
2189	1596	1633	2.6	9	12		
2191	1663	1838	2.6	12	15	15.4	17
2192	1748	1864	3	12.2	15.4	18.2	19
2193	1407	1687	2.6	13	18	19	23
2194	1419	1633	2.8	13.2	16	18.6	22
2195	1548	1864	3	13.2	15.6	18	19
2196	1996	1908	2.6	8	11.4	14.6	17
2197	1708	1633	2.2	12.4	15	17	20
2198	1448	1900	2.8	12.4	14.2	17	19
2199	1655	1838	3.4	13	19.8	22	23
2200	1984	1840	2.4	10	13.6	13	
2201	1938	1900	2.8	12			
2202	1753	1687	2.8	13.2	15.8	19.2	24
2203	1684	1840	2.4	12	14.2	17.2	19
2205	1529	1838	2.4	13.2	15.8	17.2	19
2204	1878	1838	3	13			
2206	1399	1840	2.4	10.6	14.8	19.4	
2207	1607	1840	2.4	11	14.2	15.2	17
2208	1549	1633	3	13.2			
2209	1462	1908	3	13.5	18.6	21.4	
2210	1659	1633	2.4	8	11	12.6	14
2211	1614	1687	3	13			
2212	1594	1840	2.8	13.2	16	19.4	21

2213	1971	1900	2.8	12.2	15.6	16	19
2214	1455	1908	2.8	13.4			
2215	1410	1633	2.8	12.2	15	18	20
2216	1415	1633	3	13.4	16.6	20	23
2217	1703	1633	2.8	11.4	15	18.4	21
2218	1821	1803	2.8	9.4	12.6	12.2	19
2219	1683	1838	2.2	13.2	15.6	17	20
2220	1595	1840	2.2	13	17	18	24
2221	1572	1864	3	13	17	18	25
2222	1688	1687	2.6	8.8			
2223	1468	1803	2.4	9.8	13.2	15.6	17
2224	1430	1838	2.4				
2225	1697	1838	2.8	13.2	16	18.2	21
2226	1452	1864	3	12.2			
2227	1808	1788	2.2	8.4	12.8	14.8	17
2228	1464	1838	2.2	11.2	13	14.6	19
2229	1449	1840	2.8	12.2	16	20.2	25
2230	1526	1840	3	12.2			
2231	1951	1900	3	9.6	12.2	13.4	18
2232	1375	1838	2.8	10.8	13.4	15.4	19
2233	1723	1840	3	10	12	13	18
2234	1724	1803	2.8	12.2			
2235	1547	1864	2.8	10	13.6	16	18
2236	1674	1908	2	8.2	11.2	16.4	21
2238	1609	1633	2	8.2			
2237	1873	1633	2	10.2	13.4	15.2	19
2239	1759	1788	2.4	11.8	15	19.4	23
2241	1585	1838	2.4	13.5	16	18.2	24
2240	1740	1838	2	9			
2242	1700	1687	3.4	8.8			
2243	1581	1838	2	9.8	13	16	18
2244	1546	1633	2.6	7.4	11	16	19
2245	1509	1687	3	11.2			
2246	1906	1839	3	9.8			
2247	1668	1633	2.6	9.2	12.6	14	18
2248	1668	1633	3	10.8	13	17.4	22
2249	1610	1687	2.2	10.6	13.6	18	20
2250	1661	1788	2.2	10	13.6	18.2	22
2251	1666	1633	3	10.4	14.2	16.8	22
2252	1645	1633	3.4	11.6	13.8	16.4	20
2253	1483	1788	2.8	8.4	10.2	13.6	16
2254	1618	1838	3	9	11.4	13	16
2255	1637	1687	3.4	10	13		
2256	1664	1803	2.6	9			
2257	1719	1838	2	9.4	13.4	14.2	18
2258	1874	1839	2.8	9			
2259	1843	1864	2	9			
2260	1912	1908	2	10.4	13.2	15.4	19
2261	1616	1633	3	10	13.6	14	16
2262	1672	1788	3	10.8	13.6	13.8	

2263	1897	1840	2.2	11.8	15	16.2	20
2264	1681	1633	3	10.4	13.8		
2265	1532	1839	2.6	8.6	12.4	12.8	17
2266	1843	1633	2.6	10	14	15	21
2267	1521	1687	2.6	9.4	13.4	15.4	18
2268	1568	1840	2	10	12.6		
2269	1870	1840	2.4	9.8	14.2	16.2	18
2270	1652	1856	2.8	10.4	13.6	14	15
2271	1764	1864	2.6	11.6			
2272	1747	1633	2.4	13.2	14.8	18	21
2273	1478	1908	3	11			
2274	1771	1908	2	10			
2275	1533	1687	2.8	9.2	14	14	19
2276	1662	1687	3.2	8.8	13.6	16.2	19
2277	1667	1838	2.8	7.6	11.8	18	23
2278	1656	1840	2.8	10.8			
2279	1854	1864	2	10.6	13	19.4	23
2280	1615	1788	3	13	15.8	17.8	21
2281	1976	1838	3	10.2	12.4		
2283	1523	1839	2.4	11	16.8	19	21
2282	1694	1839	2.4	10	13.8	18.6	22
2284	1619	1633	2.8	7	9.8	14	18
2285	1343	1687	2.8	11			
2286	1613	1788	3	10.8	14.6	16.8	20
2287	1465	1840	3.2	11	16.6	18.8	20
2288	1555	1856	3	10	14	20	23
2289	1945	1864	3	11.4	15	19	22
2290	1641	1838	3	12.6	16	20.8	
2291	1761	1839	2.6	9.8	13.2	13.6	
2292	1518	1840	3	9.6	11.2	13	17
2293	1886	1908	2.2	10.6			
2294	1613	1633	3	11	14.4	19.5	26
2295	1453	1838	2.8	13.6			
2296	1749	1633	2.4	9.8	13.6	14.6	18
2298	1749	1633	2.8	11.6	13.4	15	
2297	1910	1633	2.8	10	16	18	22
2299	1530	1839	3.4	11.2			
2300	1450	1633	3				
2301	1997	1788	3.6				
2302	1511	1908	2	9			
2303	1704	1803	3	6.4			
2304	1601	1840	3	11	12.2	15	17
2306	1445	1908	3				
2305	2000	1908	2	8	10.8	18	
2308	1987	1839	2.8	11	14	15.6	17
2309	1552	1908	2.8	10			
2310	1706	1840	2.6	9	12	14.2	18
2311	1693	1908	3	10.4	11.6	12.4	16
2312	1752	1803	2.6	10	12.6	15.2	17
2313	1647	1687	3	10.8			

2314	1682	1838	2.6	9.8	13.2	20.2	24
2315	1589	1633	2.6	6			
2316	1592	1788	2.8	9.2	14.8	17.2	24
2317	1657	1856	3	10.8	12.6		
2318	1539	1864	3.4	11.4	12.2	14.2	16
2319	1560	1864	3				
2320	1654	1633	2.8	12.4	14.4	19.6	22
2321	1675	1633	2.8	9	11.6	14	18
2322	1583	1856	3.4	11	12	14.6	18
2323	1673	1840	2.4	9.8	13	13.4	17
2324	1514	1856	2	9.4	12.4	19.4	24
2325	1617	1687	2.8	11	15.2	22.2	24
2326	1548	1838	2.8				
2327	1383	1839	3	8	13	14.2	18
2328	1806	1856	2.4	9.8	11	14.4	16
2329	1676	1908	2.6				
2330	1991	1633	3				
2331	1361	1788	3.4	9	10.2	14	16
2332	1992	1838	3.4	10	13.6	17.4	18
2333	1844	1839	3	11	12	13	16
2334	1651	1908	2.8				
2336	1690	1788	3.4	7.2			
2337	1650	1838	3.4	11.4	12	14.6	18
2338	1739	1633	2.4	8	11.8	14.6	17
2339	1470	1839	2.8	8			
2340	1531	1864	2.6	9.4	16.8		
2341	1686	1864	2.4	10	14		
2342	2001	1840	1.8	7.6	11.4		
2343	1437	1840	1.8	10			
2344	1851	1969	2.4	13.6	19.8		
2345	1652	1864	2.4	13.2	14.2		
2346	1540	1846	1.8	7.2	11		
2347	1385	1788	2	7	8		
2348	1628	1838	2	9.8	10.4		
2349	1982	1840	2	5.8	12.4		
2350	1960	1864	1.8	9.8			
2351	2063	1969	1.2	5.2	8.8		
2352	1849	1838	2.2	9			
2353	2024	1840	1.8	5.8	8.8		
2354	1679	1864	2	6.4			
2355	2089	1840	1.8	7.6	11.4		
2356	1580	1788	2	12			
2357	1926	1840	2	9.4			
2358	1862	1788	2.6	10.4			
2359	2028	1846	1.8	7.8			
2360	1695	1864	1.8	7.6	11.4		
2361	2007	1788	2.6	8	13		
2362	1956	1838	2	7.8			
2363	1990	1969	2	6.4			
2364	2076	1864	2	6.4			

2365	1581	2019	3.1	11.8			
2366	1637	1952	2.7	13.2			
2367	1379	2019	2.7	12.5	16.7		
2368	1703	1846	2.4	9.6	12	19	24
2370	1386	1908	3	13.2	16.8		
2369	1659	1908	2.8	11.6			
2371	1844	1908	2.7	12.6	17.5		
2372	1442	1937	2.8	13			
2373	1683	1937	2.6	11.3	12.5	18.2	22
2374	1511	2019	1.8	12	15.6		
2375	1367	1846	2.3	11.5	13	19.4	26
2376	1377	1908	2.6	11.8			
2377	1690	1952	3	13.2			
2378	1912	2017	2.2	13.2			
2379	1945	2017	2.6	9.3	10.6	16.8	19
2380	1724	2045	2.8	12.5			
2381	1984	2057	2.1	13			
2383	1529	2062	1.8	12.5			
2382	1547	2062	2.1	13.3	15.8		
2385	1478	1846	2.6	12.4	15.6		
2384	1491	1846	2.5	12.5			
2386	1870	1846	2.5	12.8	16		
2388	1452	1908	2.6	9.9	10.4		
2387	1531	1908	2	10			
2389	1607	2017	2.3	12.2			
2390	1944	2019	2.5	12.4			
2391	1552	1864	3.2	12.9			
2392	1808	1952	3.1	12.6			
2393	1468	2060	2.2	8.4			
2394	1978	2062	2.2	13.2	17.5		
2395	1688	1864	2.6	12.4			
2396	1700	1952	2.8	13			
2397	1694	2017	2	13.2			
2398	1568	2019	2.9	10	13.6		
2399	1763	2060	2.9	12.2	14.4		
2400	1663	1908	2.3	13.2	15.8		
2401	1662	1937	2.4	12.8			
2402	1648	1952	2.4	13.2			
2403	1609	2057	2.7	11.7	13.3		
2404	1910	2062	2.7	13.1	15.5		
2405	1666	1908	3	12			
2406	2043	2017	2.7	13.2			
2407	1415	2060	2.7	12.8			
2408	1526	1864	3	12			
2409	1670	1908	2.7	11	13.5	16.4	20
2410	1901	1908	2.2	11.4	12.4	17.4	22
2411	1601	1937	2.9	11			
2412	1992	1969	2.8	13.2	17.6		
2413	1752	2017	2.4	11.8	15.5	21.6	27
2414	1708	2019	2.2	10			

2415	1759	1864	2.6	13.2			
2416	1599	1908	2.9	11			
2417	1530	1969	2.5	9.8			
2420	1520	2062	2.6	13.2	13.6	17.2	21
2418	1681	2062	2.5	11			
2419	1740	2062	2.8	13.4	15.8	18.2	23
2421	1651	1846	3.1	13.2	15.4		
2422	1596	1952	3	12			
2423	1756	1969	2.1	8.2	9.5	16.4	19
2425	1848	2019	2.5	11			
2424	1897	2019	2.3	10			
2426	1455	1969	3.1	13.2	15	18	22
2427	1595	2055	2	9.6			
2428	1608	2060	2.8	12			
2429	1928	2060	2	9.3			
2431	1532	1846	3.6	12.5			
2430	1693	1846	2.8	12			
2432	1589	1864	2.6	13.2	15.9	18	24
2433	1748	1969	3.1	12.5			
2434	1936	1864	2.7	11.6			
2435	1674	1908	2.8	11.7	13.8		
2436	1548	2055	2.7	12.6	18.4		
2437	1771	2057	2.6	11.5			
2438	1672	2062	2.1	11.3			
2439	1562	2019	2.9	11.8			
2440	1656	2062	2.6	13.2			
2441	1615	1846	2.5	13.2	17.7		
2442	1605	1908	2.5	13.1	18	20.2	23
2443	1675	1952	2.2	7.7	12.4	18.2	23
2444	1747	1969	2.6	11			
2445	1450	2060	2.5	11.4	13	17.4	22
2446	1462	2062	2.7	10.8	12	17.2	20
2447	1994	1969	2	8.4	12	17.2	19
2448	1614	1864	3.1	12.5			
2449	1465	1937	3.3	13.2			
2450	1887	1937	2.6	11			
2451	1480	1969	2.8	12			
2452	1673	2017	2.5	11.5			
2453	1749	2019	2.5	11.9	15.4	19.4	23
2454	1843	2055	2.4	11.9			
2455	1483	2060	2.6	8.7			
2456	1790	1952	2.6	12.5			
2457	1699	1969	2.5	13.2			
2458	1549	2019	2.6	13.2	15.4	18	24
2459	1668	2060	2.7	13.2			
2460	1998	2017	2.5	11.5			
2461	1449	2060	2.4	10.2			
2462	1684	2019	2.4	11.9	16.5	22.4	27
2463	1383	1846	3	11.8	13.5	17	22
2464	1753	1864	3	13.2	17	20.2	22

2465	1682	1908	2.8	12.4			
2466	1873	1952	2.5	8.6	11.2	17.2	23
2467	1530	2019	2.2	9.9	11		
2468	2075	2055	2.5	12.7			
2469	1641	1846	3.1	13.2			
2470	1619	2055	3.2	12.8	15.7		
2471	1518	2060	3.2	13.1			
2472	1453	2062	2.5	12.6	16.4		
2473	1647	1846	3.1	9.6			
2474	1613	2017	2.6	9.9	14.3	18.4	21
2475	1697	2055	2.6	12.3	14.5	15.2	18
2477	1419	2019	3.1	13.2			
2476	1886	2019	2.8	10.2	11.6		
2478	2067	2017	2.2	8.4	11.4	16.4	20
2479	2030	2062	2	8.1			
2480	2089	2062	2.9	9.8			
2481	2176	2019	2.6	9.5	14	16.8	20
2482	1761	2019	3.3	12.6			
2485	1521	1952	2.3	9.8			
2483	1533	1952	2.5	9.4	14	17	17
2484	1539	1952	2.9	11.4			
2486	1961	1846	2.7	10.6			
2487	2034	1846	1.9	10	14	15.4	18
2488	1652	1908	2.6	11			
2489	1966	1969	2.9	10.7	19.1		
2490	1906	2019	2.4	7.9			
2491	1938	2017	2.4	8.9	15.8	18	21
2492	1739	2026	3	10.3	17.8	18.6	23
2493	1862	2060	3.4	8.4	14	17.8	
2494	2033	1846	2.5	10.8	12.6	14.6	
2495	2147	2019	2.9	13.4			
2496	1695	1908	2.8	12			
2497	2087	2060	2.6	11			
2499	2093	1969	2.4	12.8	16		
2498	2174	1969	2.1	11.1			
2500	1971	2019	2.7	13.4			
2501	1654	1846	2.3	5.6			
2502	2095	2153	2	10.3			
2503	2041	1846	2.1	10.2	14.1	17.8	
2504	2069	2159	2	6.7			
2505	2099	1952	3.1	13.2			
2506	1990	1969	2.4	9.3			
2507	2065	1908	2.3	10.7			
2509	1689	1969	2.4	11.9			
2508	2078	1969	2.7	13.2			
2510	2120	2017	2.6	8.3	12.7	14.6	
2511	2168	2159	2.2	10.8	14.6	19.4	
2512	2035	2017	2.3	8.4			
2513	2108	2019	2.7	10.2			
2514	2005	1952	1.4	9			

2515	2143	2060	2.1	9	10.6			
2516	1514	1846	2.7	7.3				
2517	2079	2017	3.1	12.8				
2518	2128	2017	1.8	7.9				
2519	1580	2019	1.5	6.6	10.8	14.8		
2520	2088	2026	1.7	9.8	12.2	14.6		
2522	1854	2062	1.8	6.8	14.4	19		
2521	2018	2062	2.1	7				
2523	1583	1846	2.3	11.1	15.5	18.8		
2524	1853	2019	2.8	11.9	16.2	18.6		
2525	2161	2062	2	9.5	11.6	14.8		
2526	2048	2019	2	7.8				
2527	2028	2019	2.5	9.4	16.2	18.6	20	
2528	2169	2026	1.9	7	10.8	13.4		
2529	2154	2060	2.8	8.1				
2530	2043	2062	2.2	8.2				
2532	2027	1952	3.3	12.2				
2533	2059	1969	2.2	8.7	14.2			
2534	2138	1969	2	7.4	12.3			
2535	2190	2017	2.5	8	13.5			
2536	1617	2019	2.7	12.8				
2537	2015	2019	2.8	10.3				
2538	2054	1846	2.1	6.9	12.3			
2539	2022	1952	2.7	9.3	16.6			
2540	2089	2062	2.6	13.5	16.7			
2541	1546	1969	3.1	12.4	19.3			
2542	1851	2019	2.9	11.5	17.5			
2543	1982	2062	2.4	11	11.6	14	16	
2544	2007	2017	2.8	13.3				
2545	2085	2060	2.3	8.4	13.1	16.8	19	
2546	2000	2062	3	12.8				
2547	1667	1846	2.8	13				
2548	2082	2026	2.5	9.6	10.3	14.2		
2549	2100	2060	2.4	6				
2550	2031	2060	2.2	5.6	10.7	13.8		
2551	1676	1846	2.6	10.3				
2552	1996	2019	2.1					
2554	2090	1969	1.9	5.8				
2553	2105	1969	2.3	6.2	11.2	14	18	
2555	1849	2017	2.8					
2556	1925	1846	1.9					
2558	1664	2062	2.3	11.4	15	16.4	18	
2557	2132	2062	2.3	6				
2559	1979	2017	1.9	7.9	12.8	14		
2560	2164	2017	1.9	7				
2561	2003	2019	1.9	9.6				
2562	2096	2060	2	13.2				
2563	2155	2019	2.3	9.6	13.6			
2564	1719	2019	2.4					
2565	2091	1969	2.3					

2566	1874	2017	1.9	5.4			
2567	2001	2026	2.4				
2568	2002	1846	1.9	5.5			
2569	1526	2019	3	13.2	16.6	20	21
2570	1552	2062	3.1	11.5			
2571	1562	1969	2.7	12.2	16	17.6	19
2572	1944	2019	3.1	11.4			
2573	1520	1969	2.9	13.2			
2574	1684	2017	2.9	12.4	17	22	23
2575	1992	2017	3.2	11.6			
2576	1694	2019	2.8	10.8			
2577	1984	1969	3.1	13.2			
2578	1910	2019	2.9	13.2	17.6	21.4	26
2579	1596	2218	2.8	12.4			
2580	1912	2230	2.7	11.1	13	13.8	14
2581	1897	2289	3.4	13.2	17.6	21.3	23
2582	1936	2062	2.8	13.2	16.4	18.6	21
2583	1663	2216	2.9	12.8	16.8	21	22
2584	1548	2218	2.7	12.4	15.8	19.2	20
2585	1529	1969	2.7	13.4			
2586	1929	2019	2.8	13.2			
2587	1682	2218	2.8	13.2	13.8	17.2	21
2589	1450	2289	2.8	13.3	14.8	16.2	18
2588	1637	2289	2.4	12.4	14.8	17.8	18
2590	1659	1969	2.5	12.8	15.4	20	21
2591	1945	1969	2.7	11.3			
2592	1699	2019	2.9	12.8	14.2	17.4	20
2593	1748	1969	3.3	11.1			
2594	1748	1969	2.4	13	15.2	16.4	17
2595	1700	2019	2.5	12.5			
2597	1614	2216	2.5	12.3			
2596	1961	2216	3	13.2			
2598	1693	2230	2.9	13.2	16.4	20.4	21
2599	1445	1969	3.2	13.4			
2600	1377	2062	3	12.5			
2601	1847	2165	2.9	12.2			
2602	1878	2165	3.1	11.3			
2603	1719	2218	2.8	13.2	15.8	19	22
2604	1844	2251	2.7	13.4	17.6		
2605	1848	2019	3	13.2			
2607	1759	2019	2.8	13.2	15.8	19.4	21
2606	1771	2019	2.8	13	16.2	19.4	23
2608	2176	2218	2.5	12.6			
2616	1978	2218	2.4	13.2			
2617	1668	2165	3.3	13.4			
2619	1568	2218	3	13.4			
2618	1739	2218	2.5	12			
2621	1532	2230	3.2				
2620	1688	2230	2.3	12.4	14.2	18.6	23
2622	1740	1969	2.8	13.2	15.8	19.2	22

2623	1552	2019	2.9	12			
2625	1521	2062	2.6				
2624	2027	2062	3.4	13.4			
2626	1555	2193	2.5	12.4	17.2	21.2	
2627	1697	2216	1.8	11.1	15.8	19.4	22
2628	1853	2062	2.7	13.2	18.8	19.4	21
2629	1790	2165	2.6	13.4	15	17	20
2630	1749	2193	3	12.5			
2631	1615	2230	3.2	13.2	17	19	22
2633	1533	2019	2.8	12.5			
2632	1756	2019	2.8	12			
2635	1430	2165	2.6	13.2	15	17.4	21
2634	2143	2165	2.9	13.4	14.6	18.4	21
2636	1648	2216	2.5				
2637	2034	2251	2.8	12.5			
2638	2048	2216	2.4	13			
2639	1549	2289	3	13.2	17.4	18.4	23
2640	1662	2251	2.5	13	15	16	23
2641	2041	2019	2.5	12.6	13	16.8	19
2642	1419	2165	3.2	13.2			
2643	1752	2216	2.5	12	14	18.4	20
2644	2120	1969	3.1	10.6	13.4		
2645	2168	2216	2.5	11.6	14	16	21
2646	1724	2230	2.8				
2647	1531	2289	2.7	12.2	14	16.4	20
2648	1415	1969	2.9	12.8	14.2	16	19
2650	1708	2019	2.7	12.5			
2649	1971	2019	2.5	10.6	15.4	19.4	26
2651	2033	2218	2.7	13.2	18.4	20.2	24
2652	2043	1969	3	13			
2653	1583	2019	2.8	12.6	15	18.2	22
2654	1448	2019	2.6	9.4	10.8	15.2	17
2655	1580	2216	2.6	13.2			
2656	1979	2218	2.8				
2657	2028	2230	2.8	13.4	16.2	18	22
2658	2054	2251	2.5	11.6	13.8	16.4	21
2659	1704	2289	3				
2660	1673	2289	2.2	9.6	13.2	19.2	21
2661	2037	2289	2.5	12.6	16.2	21.2	26
2662	1581	1969	2.6	9.2	14.4	18	22
2664	2046	1969	3.4				
2665	1649	2019	3.1	13	17		
2666	1982	2216	2.8	13.2	14	16.4	20
2667	1689	2289	2.7	12.5	15.8	21.8	
2668	2065	2019	3.3	10.2	12.2	15.6	18
2670	1599	2230	3	11	14	17.4	20
2669	1862	2230	2.1	8.5	9.2	12.1	18
2671	1925	2251	2.5	13.2	14.4	14.8	21
2672	1674	2218	3.6				
2673	1666	2019	2.4				

2674	2270	2193	1.8	7.8	11.4		
2676	1676	1969	3.2	13.5	16.6		
2675	1886	1969	3.1	11.5	12.2	18.5	
2677	1455	1969	3.1	10.1	12.2	16.4	21
2678	1761	2019	3	10.8	14		
2679	2083	2251	2.9	13.6	16	20.6	21
2680	1849	2062	2.2	10.5	14.8	17.2	20
2681	2063	2230	3	13.7			
2682	1695	2019	2.9	13.4	18.4		
2683	2154	2230	2.1	12.5			
2684	1966	2062	3.2	12.9	17.6	22	24
2685	1906	2193	2.3	12.7	17.2	18.8	20
2686	2059	2216	2.6	12.7			
2687	1938	2216	2.9	13.4	18.4		
2688	1956	1969	2.8	10.7	13	16	16
2689	2132	2230	3	13.1			
2690	1808	2019	2.8	12.5	18	18.6	20
2691	2007	2230	2.8	13.5	18.4		
2692	1607	2062	1.8	12.7	17.4	21.4	22
2693	2096	2165	2.7	13.3	16	20.2	19
2694	1672	2216	2.8	10.4	16.2		
2695	1703	2062	2.6	12.4			
2696	1530	2165	2.6	13.4	17.2	20.4	19
2697	2069	2165	2.8	10.2	14.2		
2698	2016	2266	1.9	12.2	18.4		
2699	2138	2319	2.8	13.2	18.5		
2700	1854	1969	1.8	11.4	13.4	16.4	21
2701	2307	2193	2	11.2	13.4		
2702	1928	2019	2.8	12.9	18.2		
2703	2031	2062	2.7	12.5			
2704	2015	2165	3	13.6	19.4		
2705	2001	2019	3.2	13.2	16.2		
2706	1655	2062	2.4	11.3			
2707	2134	2165	2.9	11.3	14		
2708	1851	1969	2.8	12.8			
2709	2035	2019	2.3	9.1	14.4	14	19
2710	2002	2193	2.9	11.2	16.2	19.4	21
2711	2067	2230	2.5	11.2	11.5	14.8	17
2712	1764	2193	2.6	13.1	16.4	18.8	21
2714	2005	1969	2.8	9.4	13	15.2	16
2713	2169	1969	3	12.7	15.4		
2715	2077	2165	2.8	12.4			
2716	1940	2216	2.4	10.7	12.8	15.6	18
2717	1694	2019	2.6	12.5	14.7		
2718	2068	2193	3.1	13	16.3		
2719	2068	2193	2.5	12.5	14.9		
2720	2007	2239	2.4	13.6	18.4	20.2	24
2721	1790	2251	2.5	13.5	18.8		
2722	1599	2019	3.5	12.5	16.3		
2723	1944	2365	3.2	11.7			

2724	1982	2019	3.1	13.2	17.8
2725	2377	2393	2.4	13.4	15
2726	1562	2365	3.1	13.3	15.8
2728	1520	2019	2.6	13.3	
2727	2041	2019	3	13.4	17.6
2729	1847	2230	3.2	12.7	17.4
2730	1704	2251	2.6	12.3	15.8
2731	1609	2251	2.6	12.1	12.5
2732	2120	2365	3.1	12.5	21
2733	1568	2193	3	13.2	
2734	1928	2216	3.1	12.4	15.6
2735	1581	2230	3.2	13.3	
2736	2016	2393	2.8	12.5	13.2
2737	1979	2193	3.2	12.7	
2738	2031	2230	3.2	13.3	
2739	1580	2239	3.5	11.8	12.6
2740	1719	2019	3.2	12.9	16.1
2741	1614	2299	3.2	12.5	
2742	2048	2395	2.9	12.7	
2743	1848	2230	3	12.7	
2744	1666	2299	3	12.4	13.4
2745	1672	2019	3	11	
2746	1749	2019	3.4	12.7	
2747	2132	2019	3.1	12.9	19.1
2748	2087	2216	3.2	13.3	17.5
2749	2143	2239	3.5	11.2	16.2
2750	1693	2251	2.7	13	
2751	2168	2283	3.3	12.5	
2754	1659	2019	2.8	13.1	
2752	2069	2019	2.8	12.8	
2753	2069	2019	3.1	12.6	14.7
2755	1870	2251	3.2	12.7	
2757	1684	2365	2.2	13.4	14.5
2756	1961	2365	2.7	13	
2759	1552	2019	3.2	13.6	
2758	1619	2019	3.1	12.6	13
2760	1984	2019	3.3	13	13.5
2761	1546	2193	3	13	16
2763	1529	2230	3.6	13.4	14
2762	2028	2230	3.2	13.4	13.8
2764	1615	2251	3.4	12.1	
2765	1949	2365	3.5	13.2	18.5
2766	2176	2393	3	12.7	
2767	1878	2395	3.4	13	18.2
2768	1596	2019	3.5	10.8	
2769	2033	2216	3.1	12.9	
2770	2155	2230	3.4	11.5	
2771	1663	2299	2.6	13.2	14.5
2772	1532	2019	3.4	13.2	
2773	1637	2283	3.5	13.3	

2774	2054	2216	3					
2775	1548	2230	3.3					
2776	1912	2239	3.2	13.2				
2777	1748	2251	2.3	9.5				
2778	1886	2299	3.7	12.7	16.4			
2779	1583	2395	2.7	12.2	15.5			
2780	1739	2216	3.2					
2781	1862	2230	2.7					
2782	1689	2251	2.9	12				
2783	1978	2299	2.9	13.2				
2785	1853	2019	3.3	13.4				
2784	2043	2019	3.2	12.5	15.9			
2786	1897	2251	3	13.5	17.5			
2787	1697	2299	2.8	12.5	15.9			
2788	2531	2019	3.4					
2789	1808	2216	3					
2790	1759	2239	2.5					
2791	1873	2251	2.8	12.5	14.8			
2792	1521	2299	2.6	11.6				
2793	2034	2019	3.4	12.9				
2794	1761	2193	2.7					
2795	2544	2217	2.9	13	16.4			
2796	1945	2239	2.5	9.7				
2797	1971	2019	2.9	13				
2798	1740	2251	3.3	13.2	16.6			
2799	1901	2019	3	12.7				
2801	2015	2251	3	12.6	14.9			
2800	2067	2251	2.3	10.6				
2802	1938	2283	2.8	13.2	17.3			
2803	2270	2395	2.8					
2804	2063	2019	2.6	12.2	15.6			
2805	1854	2019	2.1	12.5	15.4			
2806	1752	2216	3.4	13.6				
2807	1607	2365	2.5	12.6	15.7			
2808	1940	2365	2.3	8				
2809	1673	2019	2.9	12.5				
2810	1530	2251	2.6	11				
2811	2134	2283	2.9	13.4				
2812	2003	2393	2.7	10.6				
2813	2038	2251	2.4	9.6	12.6			
2814	1764	2395	2.3	9.3				
2815	1764	2395	2.5	13.2	15.8	18.6	24	
2816	2138	2395	2.7	10.2				
2817	2096	2230	3.3	12.2	12.7			
2818	2035	2239	3	11	13.4			
2819	1674	2251	2.4	12.6				
2821	2002	2019	2.5	8.9				
2820	2077	2019	3.4	13	16.4	18	18	
2822	2171	2019	3	11.5				
2823	2005	2239	1.8					

2824	1849	2251	2.8	12.8			
2825	1956	2283	1.8	7.9			
2826	2154	2365	2.6	13.1			
2827	1929	2216	2.8	10	14.7		
2828	1756	2299	2	8.2			
2829	2543	2393	2.3	8.1			
2830	1703	2019	3.2	12.8			
2831	2553	2283	2.2	7			
2832	2335	2019	1.2	5			
2833	1555	2393	2.6	9.1			
2834	2270	2218	2.7	12.6	15.2		
2835	1990	2239	3.1	12.5	15.2		
2836	1851	2218	3.6	13	17	22	26
2837	1526	2017	2.9	13.3	17.4	21	24
2838	1699	2216	3.6	11.2	14.4		
2839	1695	2239	2.9	13.1	18.8	21.8	26
2840	1682	2251	2.1	13.4	16.8	19.8	24
2841	1966	2017	2.6	12	15		
2842	2649	2165	2	10.4	15.4		
2843	1708	2251	2.5	13.5	15.8	19.8	23
2845	2001	2165	2.1	9.3	12		
2846	2307	2193	2	9.3			
2847	1688	2251	2.7	11.5			
2848	1724	2251	3.1	11.7	15.8		
2849	2169	2017	2.5	12	15	19.8	19
2850	2592	2216	2.6	11.3	18.4		
2851	2577	2273	2.1	9.6	15.2		
2852	2663	2251	1.9				
2853	2584	2273	1.8	8	13.2		
2854	2677	2193	2.3	10.2	15.8		
2855	2586	2193	2.8	9.2	14.8	16.8	18
2856	2569	2216	1.7	9	13.2	15.6	18
2857	2574	2216	1.7	8.3	16.8		
2858	2658	2273	1.6	10.8	14.4	14.1	
2860	2531	2365	2.3	8.2	13.4	14.9	16
2859	2662	2365	2.1	9.8	14.2	16.2	18
2861	2642	2216	2	11.2	12.4		
2862	2556	2273	2.7	9.5	18		
2863	2588	2193	2.1	10	14.6		
2864	2671	2218	2.6	10.9	19.2		
2866	2640	2251	2.3	10.4	15	16.3	20
2865	2685	2251	2.1	11.8	18.6	20.6	22
2867	2651	2283	2.4				
2868	2046	2251	3.2	12.5	17.5	21.3	25
2924	2132	2218	3.2	11.2	16.5		
2925	1614	2251	3	10.8			
2926	1862	2217	3.1	11.6			
2927	2120	2230	2	8.6			
2928	2120	2230	2.1	12.5			
2929	2143	2216	3	13.5			

2930	1886	2289	3	13			
2931	1961	2230	3.2	12.6	17.5	19	25
2932	1961	2230	2.1	12.6	15.6		
2933	1910	2251	2.1	13			
2934	1979	2251	3.4	11.5			
2935	2176	2218	2.9	12.4			
2936	1982	2273	2.5	12.5	16.3		
2937	1925	2273	2.6	13.3	14.1		
2938	2034	2289	3	12.4	13.9	15.6	22
2939	2033	2610	3.1	13.5			
2940	2077	2611	2.5	13.6			
2942	1759	2193	2.7	12.5	15.8	18.1	23
2941	1929	2193	3.1	13			
2943	1663	2251	2.8	12.4			
2944	1912	2251	2.9	11.3	12.9	15.9	20
2945	2054	2193	2.7	12.5			
2946	1901	2193	2.7	11.8	14		
2947	1703	2218	2.3	11.7			
2948	1853	2218	2.9	12.5			
2949	2335	2216	2.5	10.2	12.9		
2950	2087	2218	2.3	13.6			
2952	2048	2251	3.2	13.1			
2951	2168	2251	3.2	13			
2953	2035	2289	2.2	11.5	13.6	14.8	19
2954	2068	2193	3	13			
2955	2068	2193	3.1	13.2			
2956	2067	2216	2.3	12.4	13.9		
2957	1928	2217	2.4	13			
2958	2069	2230	3.6	13.2	17		
2959	2083	2251	2.7	12.5			
2960	1971	2193	2.7	10.2	12.2	15.6	18
2961	2028	2218	2.9	13.5			
2963	2005	2193	2.4	13.5			
2964	2134	2218	2.9	13.5			
2965	2138	2273	2.8	13.4			
2966	2377	2611	2.5	8.9	12.1		
2967	1532	2193	3.2	10.5	12.9		
2968	1682	2251	2.5	12.5	15.3		
2969	1609	2289	2.7	11.4	14.1	15.7	21
2970	2096	2611	2.8	13.6			
2971	1854	2193	2.6	11.8	14.7	21.4	24
2972	1992	2218	1.8	11.6	13.9		
2973	1992	2218	2.3	10.8			
2974	1764	2193	2.3	9.6			
2975	2171	2218	3	11.5			
2976	1897	2251	2.6	10.5			
2977	1949	2251	2.2	10.9	12.4		
2978	1956	2230	2.8	9.8	11.8	13.4	16
2979	1878	2193	2.5	10.4	13.4	16	19
2980	2031	2251	3.3	11.5			

2981	2169	2273	2.7	11.4	13	
2982	1739	2230	2.5	8.5		
2983	2465	2612	2.5	12.7	14	
2984	2043	2609	2.7	9.8	14.4	
2985	2046	2615	2.9	13.2	17.8	
2986	2457	2613	2.8	13.5	18.1	
2987	2002	2612	2.7	11.5	12.5	
2988	2059	2614	2.7	9.3	10	
2989	2805	2614	2.4	9.5	11.4	
2990	2001	2614	2.4	11.6	13.5	
3086	2858	2905	2.8			
3087	2979	2907	3.1			
3088	2856	2907	2.5			
3092	2984	2900	2.2			
3093	2939	2901	1.5			
3094	2859	2901	2.5			
3095	2949	2908	2.7			
3096	2933	2903	2.6			
3097	2855	2902	2.9			
3098	2978	2907	2.2			
3099	2962	2900	2.4			
3115	3080	2899	2.1	8.1		
3116	3076	3001	1.9	7.3		
3117	3077	3005	2.9	11.2	17.2	21
3118	3073	3004	2.5			
3119	3075	2997	2.6			
3120	3079	3005	1.5			
3121	2984	3083	3.2			
3122	2874	3091	2.3	12.9		
3123	2890	3091	2.7	11		
3124	2897	3091	2.4	13.4		
3125	2878	3005	3.2	13.2		
3126	2933	3000	3	13.2		
3128	2886	3083	3.2	13.7		
3127	2892	3083	2.6	12		
3129	2991	3083	3.1	13		
3130	3034	3090	1.9	8.2		
3132	3020	2906	2.7	12.3		
3131	2875	3084	2.8			
3133	2989	3113	2.7	13		
3134	2941	3083	2.6	12.5		
3135	2962	3113	2.4			
3137	2882	3005	2.4	12.6		
3136	2869	3083	3			
3138	2978	2899	2			
3141	3015	3005	1.8	12.6		
3139	2885	3085	2.2	8.2		
3140	3058	3113	3.2			
3142	3069	2899	2.2			
3143	2881	2906	2.3	12.7		

3145	2893	2906	1.2	
3144	3046	3085	3.1	
3146	2880	3085	2.6	
3147	2992	3091	3	10.5
3149	3049	2898	2	
3148	3093	3085	2	
3150	3062	3081	2.3	11.6
3151	2960	3090	2.1	9.7
3152	2995	3090	2.4	12.5
3153	3006	3089	2.1	13.6
3154	3006	3089	2	
3156	2870	2899	2.6	11.8
3155	2873	3085	3	
3158	2871	2997	3	10.3
3157	2968	3089	2.9	
3159	3032	2906	2.1	
3160	2895	3090	3.1	10.7
3161	3043	3091	2.6	
3162	2884	2919	2.4	
3163	2887	3083	3	
3164	3007	2914	3.3	13.5
3165	3047	2844	3	13.5
3166	2994	2909	2.9	12.9
3167	3077	2922	2.6	12.1
3170	3052	2904	1.9	8
3171	3027	2914	3.5	9.2
3168	3010	2971	2.6	11.2
3169	3056	2998	2.7	11.1
3172	3088	2914	3	13
3173	3040	2922	2.8	12.6
3175	2888	2904	2.9	12.5
3174	3039	2971	3.1	13.4
3176	2888	2909	2.2	10.7
3177	3071	2971	3	11.1
3178	2996	2998	1.9	11
3179	2996	2998	2	11.8
3180	3019	2914	3.2	13
3181	3033	2914	3	
3182	2855	2971	2.9	
3183	2879	2844	2	
3184	3067	2914	2.5	
3186	2872	2914	2.5	
3185	2993	2971	2.6	
3187	3021	2844	2.9	
3188	2896	2914	3.1	
3189	3017	2914	3	
3190	3036	2971	2.4	
3191	2889	2918	3.2	
3192	3011	2844	2.5	
3193	3020	2914	2.6	

3195	3070	2914	2.8				
3194	3044	2998	2.7				
3196	2891	2909	3.1	11.9	17.3	21.7	
3197	3029	2914	2.6				
3198	3008	2971	2.7				
3199	3064	3002	2.3	11.8	15	17.9	
3200	3034	2909	2.8	13.5			
3201	3016	2917	2.5				
3203	3037	2909	2.5				
3202	3073	3090	3	10.5			
3204	2878	2998	2.9	13.4	20.8	24.4	27
3206	3030	3003	2.7	12.8	18	20.2	
3205	2876	3003	2.9	12.5			
3208	2995	2918	2.7				
3207	2933	3002	3.1	13.5			
3209	2892	2909	1.3				
3210	2892	2909	2.5				
3213	3041	2909	2.3	9.1	12.4	13.3	18
3214	2869	2909	2.4	8.7			
3215	3009	2917	2.1	9.9			
3212	3015	2998	2.3	11.1	14.8	16.9	
3211	3054	2998	2.7				
3216	2886	3002	3.1	11.9			
3218	3065	2909	3.1	13.4	18.9	24.5	27
3217	3059	3003	3	8.4			
3219	2871	2909	3.4	10.4	16.1	18.2	
3220	2984	2909	3.4	13	19.1	25.1	
3222	3013	2918	1.4	7.5			
3223	3013	2918	1.4	7.8			
3221	3066	2918	2.9				
3224	3049	2918	2.6	12.1	16.6		
3226	3048	2917	2.3				
3225	3026	3002	2.5	12.6			
3230	3068	2909	2.4	8.3			
3227	3012	2998	3	11.2			
3229	3018	3003	1.9	9			
3228	2870	3003	2.9				
3232	3051	2909	2.4	10.2			
3231	3053	2998	3	13.5			
3233	3006	2917	2.9				
3234	2941	2918	2.7	10.4	15		
3235	3038	2917	2.2	12			
3236	2881	2918	2.3	10.3			
3239	2992	2909	2.4	9.5	16.3		
3240	3093	2918	3	8.8			
3237	3072	2998	2.7	7.7			
3238	3043	3003	2.6	11.6			
3242	3058	2998	2.7				
3243	3024	3002	2.8	9.2			
3241	3022	3091	2.6	8.9	14		

3244	3063	2909	2.9	10.8	16.7	21.7	25
3245	3079	2917	2.5				
3247	2877	2917	3.2				
3246	2858	3091	2				
3250	2962	2917	2.6	9.7			
3248	2968	3003	2.9	10.2			
3249	2968	3003	2.2				
3251	3055	2998	2.8				
3253	3080	2909	2.6				
3252	3060	2998	2.6	9.8	15.2		
3254	2988	2917	2.9	11.3	16.1		
3255	2890	3082	2.8				
3256	3076	2909	3.1				
3257	3078	2917	2.9				
3258	2874	2909	2.8	13.2	18.2	24.1	
3262	3014	2917	2.7	10	16.8	21.4	26
3261	2883	2917	2.4	5.7			
3263	2883	2917	1.8				
3260	3028	3003	2.1				
3259	2897	3003	2.6	7.3			
3264	3050	3091	2.2				
3265	2882	3003	2.7	8.2	16.7	21.1	27
3266	2991	3081	3.4				
3267	2880	3111	2.3				
3270	2885	2917	1.8	12.6			
3268	3036	3101	1.9	12.1	16.4	16.4	18
3269	3067	3102	3	11.2			
3272	3074	2917	3.3	13	18.1	20.2	24
3271	2855	3107	2.8	10.2	13.2		
3273	3052	2916	3.3	12.3	15.1	17.2	20
3274	3007	2923	2.1	13.1			
3275	2887	3109	3	13			
3276	3077	2920	3.4	13	18.8		
3279	2888	2911	2.5	13.6	16	19	23
3277	3071	3106	2.6	13			
3278	3020	3108	2.6	13.6			
3280	3039	2913	2.6	13.5			
3281	3035	3110	2.3	13.6	16.2	18	22
3282	3062	2913	2.1	12.7			
3283	2875	3102	3	10.8	14.5		
3285	3025	2923	2.4				
3284	3045	3103	1.9				
3287	2978	2913	2.6				
3286	2879	3107	3	13.1	15.8		
3288	3057	3103	2.9	11.1	15.2	18.7	19
3289	3075	3101	3.2				
3290	3069	3109	2.5				
3292	2996	2917	2.8				
3293	2884	2923	3.3				
3291	2896	3102	2.6				

3294	3042	2920	3.1				
3295	3088	3102	2.4				
3296	3070	2916	3.4				
3297	2889	2917	2.6				
3298	3011	2923	2.8				
3299	2933	2910	3	11			
3300	3048	2913	2.8	10.8			
3301	2941	2911	2.1	10.1			
3302	2941	2911	2	12.9			
3303	3031	2915	2.8	12.4			
3304	2878	2917	2.9	13.5			
3305	2886	2915	2.8	13.5			
3306	2891	2911	2.6	13.6			
3307	3112	2921	2	13.5			
3308	3026	2913	2.9	13.5			
3309	3080	2913	2.8	12.8			
3310	3076	2913	2.8				
3311	2994	2913	2.9	7	9.4		
3312	3046	2912	2.4				
3313	3063	2917	3	10.2			
3314	2882	2911	2.6				
3315	3037	2917	2.7	13	16.7	18.9	18
3316	2895	2999	2.8				
3317	2876	2999	2.9	12.2			
3318	3066	2917	2.2	10.4			
3319	3022	2913	2.7	12.7			
3320	3079	2915	2.5	10.9			
3321	3079	2915	1.9				
3322	3050	2921	2.7	13.2			
3323	3059	2910	3.6	11.1			
3324	2870	2910	2.6	10.7			
3325	2877	2911	2.5	13.4			
3326	3049	2910	2.6	11.3			
3327	3065	2911	2.6	12			
3328	3019	2921	3.3	13.2			
3329	2991	2915	2.8	12			
3330	3013	2917	3.4	12.5	16.5	19.5	19
3331	3054	2913	2.8	12			
3332	2993	2915	3.2	10.1	14.6	17.5	23
3333	3008	2910	2.9	13.2	15.2	17.2	17
3334	2992	2912	2.8	8.4			
3335	3023	2915	2.7	12.8			
3336	2894	2910	3	13			
3337	2995	2911	3.1	10.9			
3338	3053	2913	3.3	13			
3339	3014	2999	2.8	12.3	14.6	16	16
3340	3061	2999	2.5	12.3			
3341	2869	2910	2.5	10.5			
3342	3044	2911	2.7	9			
3345	3056	2911	2.6	10.4	13.7	18.8	22

3343	2883	3104	1.8	8.7			
3344	2883	3104	2.1				
3346	2988	3100	2.6	12.6			
3348	2881	2921	2.6	12.8			
3347	3051	2999	2.5	12.1	16.4	21.6	20
3349	3072	3107	3.7	13.5			
3350	3029	3108	3.3	12.1			
3351	2871	2913	2.8	12.4			
3352	3073	3109	2.7	11.4			
3353	3040	2910	3.1	11.6			
3355	2874	2911	2.5	10.6			
3354	3078	3106	2.9	10.1			
3356	3058	2913	2.8	11.5	13.1	14.1	15
3357	3006	2999	2.3	10.4			
3358	3006	2999	2.4	10.9			
3359	3114	3105	2.2	6.9			
3360	2962	2915	3	10.9			
