# Supplementary material for: Development of selection strategies for genetic improvement in production traits of Mecheri sheep based on a Bayesian multi trait evaluation
Source: PLoS One. 2023 Dec 14;18(12):e0289460. doi: 10.1371/journal.pone.0289460 (PMC10721189; doi:10.1371/journal.pone.0289460)
Supplement: Attachment — Submitted filename: Response to Reviewers.docx [file pone.0289460.s002.docx]

**Response to Reviewers**

We thank the academic editor and reviewers for the review and decision on regarding our submitted manuscript entitled, ‘Development of selection strategies for genetic improvement in production traits of Mecheri sheep based on a Bayesian multi trait evaluation’ (Ref. No. PONE-D-23-22558).

**Response to Academic Editor’s comments (Journal Requirements)**

**Review point 1. PLOS ONE's style requirements**

**Response**:

- Foot notes and legends under the tables were corrected as per the journal’s style.
- Figure title and legends were corrected as per the journal’s style
- Equations were recreated with equation tools (MS word) as inline equations
- Mistakes in citing the order of the Reference within the text,[38] and [37] are corrected as the order of [37] and [38]
- Supporting information is included as per the style of the journal.

**Review point 2. Language usage, spelling, and grammar**

**Response**:

The grammar, spelling, and usage of language throughout the entire document have been carefully reviewed and edited. The 'Revised Manuscript with Track Changes' file has corrections marked up. Dr. Moses Okpeku, who is from a native English-speaking nation, has reviewed the content.

**Review point 3. Funding for this work**

**Response**: We wish to mention that “the authors received no specific funding for this work”.

**Review points 4 & 5. Data sharing**

**Response**: Data used in the analysis is anonymized and submitted as supporting information file along with the manuscript (S1_File).

**Review point 6. Captions for Supporting Information files**

**Response**: Caption for the Supporting Information file is included at the end of the manuscript.

**Review point 7. Reference list review**

**Response**: References in reference list and text have verified for their completeness and correctness.

**Response to Reviewers’** **comments**

| **Reviewers comments** | **Response** |
| --- | --- |
| Reviewer #1: the paper title as well as the objective is very interesting. The methodology part was incorporated with wisely and correctly keep it up. statistical analysis part were better to do by use latest software. | The authors are thankful for the suggestion. We used the software program GIBBS2F90 in the data analysis and trust that this software is robust and relevant to the current study. |
| Reviewer #2: Numbers of records used for the analysis should be clear in the abstract and methods sections – Please incorporate in abstract. Although Table 1 has the numbers of records it is not clear if all the lamb records have an identified dam and sire (do all lambs have identical dam and sire? If not, can we create a different table with number of lambs from the different sire (sire 2, sire 2 etc) and Dams (Dam1, Dam 2 etc)?. It would be clearer to the reader if "Dams (n=) and their progeny (n=) were analyzed. All lambs had identified sires (n=)" (Same as above). | We thank the reviewer for critical assessment of the manuscript and their comments and the number of lambs with identified sires and dams, and number of dams and sires used to produce the lambs were included in the first paragraph of materials and methods section.  The number of lamb records used were 2616, 2286, 1578, 1203 and 1019 for BW, BW3, BW6, BW9 and Bw12 respectively which is sired by 226, 208, 183, 167 and 160 rams for BW, BW3, BW6, BW9 and Bw12 and the number of dams belonging to the lambs records were 1044, 961, 814, 701 and 638 respectively. |
| Table 4 - model 2 is missing as is indication of best fit. | The authors are grateful for the observation and the Model 2 has now been correctly entered in Table 4 |
| Discussion "birth weight" is birth weight a "growth factor"? For clarity suggest "birth weight is usually measured and used as an indication of growth potential" | The authors are grateful for this suggestion which has been effected accordingly, - Line number 239 |
| Discussion "post-weaning growth traits" mid-paragraph. Suggest "Research on growth traits has established age-related increase in direct heritability..." Last sentence of the paragraph is long suggest breaking to two sentences. "The present study estimated moderate heritability values for post-weaning growth traits. This demonstrates there is adequate genetic variability for improved post-weaning growth through genetic selection." | The authors appreciate the very useful suggestion, which has been effected accordingly, - Line numbers 296-303. |

(A.K.THIRUVENKADAN)
